# Engineering Cell Membrane‐Cloaked Catalysts as Multifaceted Artificial Peroxisomes for Biomedical Applications

**DOI:** 10.1002/advs.202206181

**Published:** 2023-04-25

**Authors:** Dongmei Yang, Yuanjiao Tang, Bihui Zhu, Houqing Pang, Xiao Rong, Yang Gao, Fangxue Du, Chong Cheng, Li Qiu, Lang Ma

**Affiliations:** ^1^ Department of Ultrasound Frontiers Science Center for Disease‐Related Molecular Network West China Hospital Med‐X Center for Materials Sichuan University Chengdu 610041 China; ^2^ College of Polymer Science and Engineering State Key Laboratory of Polymer Materials Engineering Sichuan University Chengdu 610065 China; ^3^ Department of Ultrasound West China Second University Hospital Sichuan University Chengdu 610041 China

**Keywords:** artificial peroxisome, biomedical application, catalyst, cell membrane, reactive oxygen species

## Abstract

Artificial peroxisomes (APEXs) or peroxisome mimics have caught a lot of attention in nanomedicine and biomaterial science in the last decade, which have great potential in clinically diagnosing and treating diseases. APEXs are typically constructed from a semipermeable membrane that encloses natural enzymes or enzyme‐mimetic catalysts to perform peroxisome‐/enzyme‐mimetic activities. The recent rapid progress regarding their biocatalytic stability, adjustable activity, and surface functionality has significantly promoted APEXs systems in real‐life applications. In addition, developing a facile and versatile system that can simulate multiple biocatalytic tasks is advantageous. Here, the recent advances in engineering cell membrane‐cloaked catalysts as multifaceted APEXs for diverse biomedical applications are highlighted and commented. First, various catalysts with single or multiple enzyme activities have been introduced as cores of APEXs. Subsequently, the extraction and function of cell membranes that are used as the shell are summarized. After that, the applications of these APEXs are discussed in detail, such as cancer therapy, antioxidant, anti‐inflammation, and neuron protection. Finally, the future perspectives and challenges of APEXs are proposed and outlined. This progress review is anticipated to provide new and unique insights into cell membrane‐cloaked catalysts and to offer significant new inspiration for designing future artificial organelles.

## Graphical abstract

Recent progress in engineering cell membrane‐cloaked catalysts as multifaceted artificial peroxisomes (APEXs) for diverse biomedical applications is summarized in this review. The reactive oxygen species‐based biomedical strategies to kill pathogenic cells or protect normal cells, such as cancer therapy, antioxidant, anti‐inflammation, neuron protection, and primary challenges and perspectives are carefully outlined, which will provide new horizons for further development of APEXs.

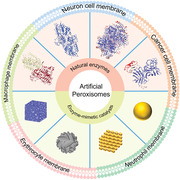

## 1 Introduction

Peroxisome, one of the important organelles in most eukaryotic cells, is surrounded by a single membrane and is filled with a variety of natural enzymes, such as oxidase (OXD), peroxidase (POD), catalase (CAT), and superoxide dismutase (SOD) that are necessary participants in biological growth and cell metabolism, which dominates the metabolism of fatty and amino acids, and the regulation of O_2_ and reactive oxygen species (ROS).^[^ ^1^, ^2^, ^3^, ^4^, ^5^ ^]^ Recently, natural enzymes have attracted more and more attention in the biomedical field due to their inherent non‐toxicity and biocompatibility.^[^ ^6^, ^7^, ^8^, ^9^ ^]^ However, the limitations of natural enzymes, such as low stability, high cost, troublesome preparation, and difficulty in recycling, prevent the potential and wide applications. Artificial peroxisomes (APEXs) or peroxisome mimics have caught much attention in nanomedicine and biomaterial science in the last decade, which have great potential in clinically diagnosing and treating diseases.^[^ ^10^ ^]^ The emergence of enzyme‐mimetic catalysts has improved the above problems of natural enzymes and changed the landscape of APEXs.^[^ ^11^ ^]^ Enzyme‐mimetic catalysts are catalytically active macro‐/nanomaterials such as inorganic metal/metal oxide and metal–organic framework (MOF) nanoparticles that can mimic natural enzymes with similar catalytic activities,^[^ ^9^, ^12^, ^13^, ^14^ ^]^ which have been widely used in molecular detection, environmental monitoring, and disease diagnosis and treatment because of their superior catalytic activity, high stability, low cost, and other characteristics compared with natural enzymes.^[^ ^8^, ^15^, ^16^ ^]^

Although enzyme‐mimetic catalysts have many advantages in biomedical fields, there are still tremendous problems with constructing APEXs that impede their further wide applications. Once enzyme‐mimetic catalysts are administered into the body, they are recognized by the immune system and uptake by the mononuclear macrophage system, leading to shortened circulation time and insufficiently targeted accumulation.^[^ ^17^, ^18^, ^19^ ^]^ Many efforts have been made to improve these deficiencies. And it is important to encapsulate these enzyme‐mimetic catalysts into one nanovesicle of polymers or lipid bilayer. For example, polymeric shells such as polyethylene glycol (PEG) are woven on the surface of natural enzymes through physical absorption or chemical conjugation, but their usage has been limited in vivo due to their low biocompatibility and high immunogenicity.^[^ ^20^ ^]^ In comparison, wrapping catalysts with natural vesicles from cell membranes can significantly enhance the delivery of catalysts. On the one hand, this cell membrane cloaking nanotechnology does not affect the catalytic activity of the original catalysts.^[^ ^21^ ^]^ On the other hand, after different cell membrane cloaking, catalysts can be endowed with these biointerfacing properties by cloaking specific cell membranes with desired functions like an immune escape, long circulation, and homologous targeting ability onto them.^[^ ^20^, ^22^, ^23^, ^24^ ^]^ Thus, intelligent APEXs based on cell membrane‐cloaked catalysts were designed and developed to diagnose or treat various diseases such as tumors and oxidative stress injuries.^[^ ^25^, ^26^, ^27^ ^]^

**Scheme 1:**
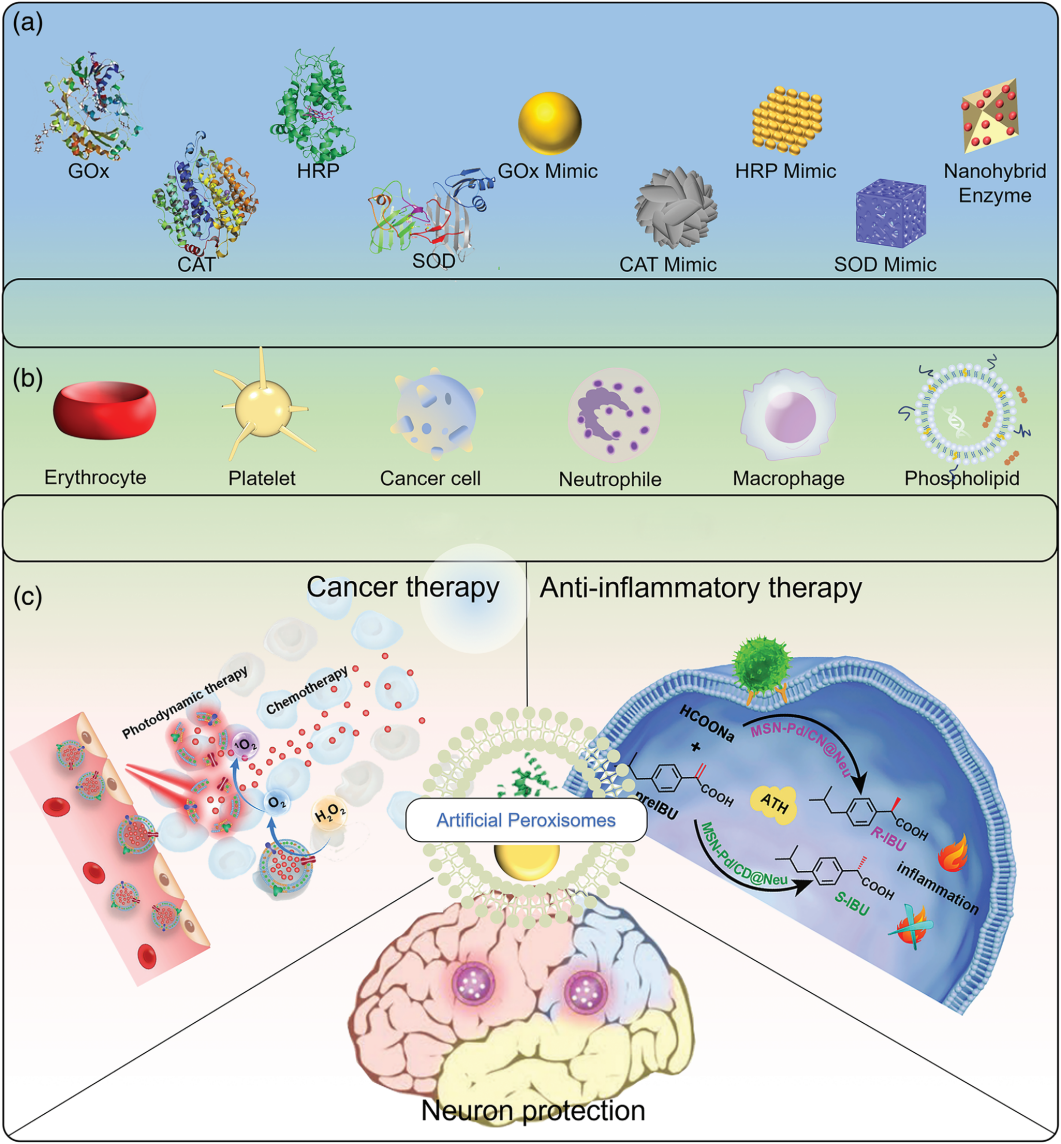
Illustrations of APEXs for diverse biomedical applications. a) Catalyst cores. Reproduced with permission.^[16]^ Copyright 2021, Wiley‐VCH. b) Source of cell membranes. Reproduced with permission.^[28]^ Copyright 2020, Elsevier Ltd. c) Representative biomedical applications. Reproduced with permission.^[29]^ Copyright 2019, Wiley‐VCH.

Here, in this timely review, we presented the most recent progress in engineering cell membrane‐cloaked catalysts as multifaceted APEXs for diverse biomedical applications, including eukaryotic cell membranes, phospholipid membranes, representative natural enzymes, and enzyme‐mimetic catalysts (**Scheme** **1**). And the ROS‐based biomedical strategies to kill pathogenic cells or protect normal cells, such as cancer therapy, antioxidant, anti‐inflammation, and neuron protection, are discussed in detail. First, we highlighted the types and synthetic strategies of both natural enzymes and enzyme‐mimetic catalysts. Then, the extraction methods and characteristics of various cell membranes are described in detail. In addition, the preparation processes, and biomedical applications of the APEXs based on cell membrane‐cloaked catalysts are summarized and discussed in detail. Finally, potential challenges and development prospects of APEXs based on cell membrane‐cloaked catalysts are covered. We believe that this review will provide new horizons for the further development of catalysts and APEXs and promote their transformation from laboratory to clinic.

## 2 Natural Enzymes for APEXs

Natural enzymes can facilitate various biological reactions occurring in living organisms.^[^ ^30^ ^]^ Natural enzymes are proteins composed of amino acids with the advantage of high selectivity and catalytic activity under moderate ambient conditions. Natural enzymes have also been reported for the diagnosis or treatment of disease due to their non‐toxicity and good biocompatibility.^[^ ^31^, ^32^ ^]^ In this section, we focus on the characteristics and applications of several representative natural enzymes, such as glucose oxidase (GOx), horseradish peroxidase (HRP), CAT, and SOD.

**Figure 1:**
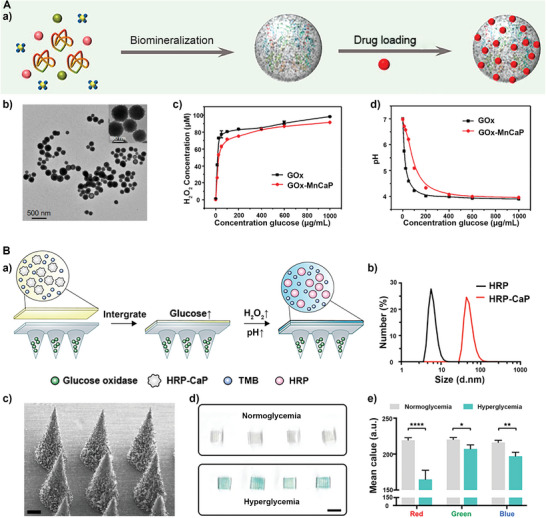
A) a) Synthetic process schematic illustration and b) transmission electron microscope (TEM) image of DOX‐loaded GOx‐MnCaP. c) The level of H_2_O_2_ produced by GOx and d) pH values after co‐incubation with glucose. Reproduced with permission.^[^ ^35^ ^]^ Copyright 2019, American Chemical Society. B) a) Schematic diagram of the microneedle sensing platform for hyperglycemia sensing. b) Particle sizes of HRP and HRP‐CaP. c) The morphology of the microneedle sensing platform by scanning electron microscope (SEM), scale bar = 200 µm. d) Scanned images and e) RGB mean value of the colorimetric microneedle patches in mice with normoglycemia (100 mg dL^−1^) and hyperglycemia (400 mg dL^−1^). Reproduced with permission.^[^ ^38^ ^]^ Copyright 2020, Elsevier Ltd.

GOx is a type of endogenous oxidoreductase that is widely distributed in living systems.^[^ ^33^ ^]^ The glucose is catalytically oxidized by GOx under oxygen into H_2_O_2_ and glucose acid. This catalytic reaction process could be used to diagnose or treat diseases, for instance, GOx‐based biosensors were used for cancer diagnosis or monitoring glucose levels of diabetic patients. Recently, a hyaluronate‐gold nanoparticle/GOx complex was reported, which could be used for monitoring glucose levels in sweat.^[^ ^34^ ^]^ Additionally, GOx‐mediated glucose consumption could be used for cancer starvation therapy. However, the insufficient stability of GOx limits its further application. A nanoparticle by mineralizing GOx with manganese‐doped calcium phosphate (GOx‐MnCaP) was prepared by an in situ biomimetic mineralization method, then loaded with doxorubicin (DOX) (**Figure** **1**A).^[^ ^35^ ^]^ The biomineralization strategy could furthest retain the activity of GOx and effectively improve its stability of GOx.

POD is another essential nature enzyme, which is also widely used in biomedicine, and HRP is one of them. HRP, a type of glycoprotein found in plants, catalyzes the oxidation of aromatic substrates and produces hydroxyl radicals in the presence of H_2_O_2_.^[^ ^36^ ^]^ HRP has been widely used as biochemical detector and sensor in clinical trials. For example, antibody–biotin–streptavidin–HRP can be used as a sensitive method for detecting fumonisins in food.^[^ ^37^ ^]^ To develop a more efficient sensor, researchers have combined two enzymes to form a dual enzymatic system. The Gu group developed an all‐in‐one sampling and displayed a transdermal colorimetric microneedle patch for sensing hyperglycemia in mice (Figure 1B).^[^ ^38^ ^]^ The cascade enzymatic reaction of GOx and the HRP‐CaP at abnormally high glucose levels triggered coloration of 3,3′,5,5′‐tetramethylbenzidine (TMB) (Figure 1Bd,e).

**Figure 2:**
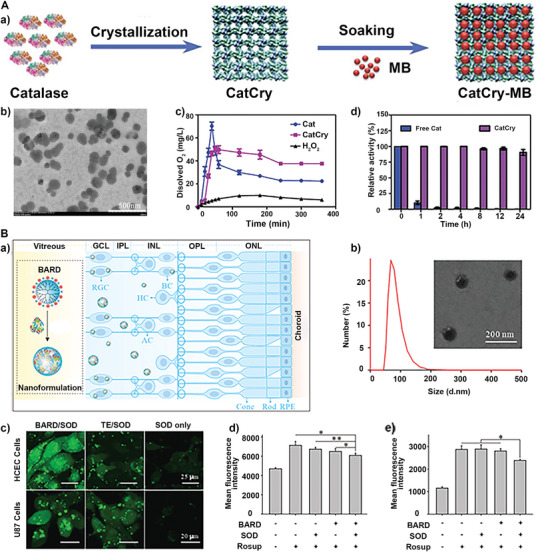
A) a) The preparation process and b) TEM images of CatCry‐MB. c) Generated O_2_ levels and (d) relative activity of free CAT and CatCry after incubation with H_2_O_2_. Reproduced with permission.^[^ ^41^ ^]^ Copyright 2021, Wiley‐VCH. B) a) Schematic diagram of intravitreal injection of BARD/SOD nanoformulations to save the retina after the ischemic injury. b) Dynamic light scattering size measurements and TEM of BARD/SOD. c) Fluorescence images of HCEC and U87 cells after different treatments for 6 h, respectively. ROS levels of d) HCEC and e) U87 cells after BARD and SOD treatment. Reproduced with permission.^[^ ^44^ ^]^ Copyright 2021, Elsevier Ltd.

In contrast to GOx and POD, some antioxidant enzymes could scavenge ROS, such as CAT as an antioxidant enzyme catalyzing the decomposition of H_2_O_2_ into O_2_ and H_2_O for the supplementary of cells and tissues, which prevents the ROS‐related vital biomolecule and body tissue damage.^[^ ^39^ ^]^ Therefore, CAT can be used to alleviate ROS‐related damage, such as inflammation, stem cell protection, and bone regeneration.^[^ ^40^ ^]^ Additionally, to regulate the hypoxic microenvironment in tumors, a photodynamic therapy (PDT) drug platform (CatCry‐MB) was constructed, in which CAT nanocrystals (CatCry) served as in situ oxygen‐generating systems and were loaded with photosensitizer methylene blue (**Figure** **2**Aa).^[^ ^41^ ^]^ CatCry, with high stability and recyclable catalytic activity, could decompose endogenous H_2_O_2_ for a long time to produce O_2_ (Figure 2Ac,d) to enhance PDT. A DOX‐loaded liposome‐holo‐Lf nanocomposite (Lf‐Liposome‐DOX) was developed, in which holo‐Lf is holo‐lactoferrin and could catalyze the decomposition of H_2_O_2_ into O_2_. Liposomes can increase the stability of CAT.^[^ ^42^ ^]^ Thus, the engineered Lf‐Liposome‐DOX nanocomposites could be used to relieve cancer hypoxic microenvironment as evidenced by photoacoustic imaging (PAI) and enhance radiochemotherapy. Similar to CAT, the SOD is also an antioxidant enzyme that can prevent the oxidative damage of proteins and DNA by removing free radicals or reactive species. As known, ·O_2_^−^ can cause cardiovascular diseases, including atherosclerosis and ischemia‐reperfusion injury. SOD can dismutate ·O_2_^−^ to H_2_O_2_ and O_2_.^[^ ^43^ ^]^ The high level of SOD in retinal cells provides a promising strategy to reduce oxidative stress and prevent retinal ischemia/reperfusion (I/R) injury. However, realizing stable intracellular antioxidant enzyme delivery remains a challenge. An SOD nanoformulation was developed, SOD assembled with boronic acid‐rich dendrimer, which could efficiently deliver SOD into retinal cells and protect the function of the retina in both in vitro and in vivo experiments (Figure 2Ba).^[^ ^44^ ^]^ The hydrodynamic size of SOD co‐assembled with boronic acid‐rich polymer (BARD) is 100–200 nm (Figure 2Bb). As shown in fluorescence images of the tissue sections, BARD efficiently delivered SOD into human corneal epithelial cells (HCEC) and human glioma cells to reduce intracellular ROS levels (Figure 2Bc–e).

As mentioned above, though with high catalytic activity and selectivity, many natural enzymes and biomacromolecules suffer from abundant intrinsic limitations.^[^ ^45^ ^]^ Researchers have improved the stability of native enzymes through biomineralization, liposomal coating, and the binding of dendritic molecules. Still, the high cost, difficulties in recycling, and other shortcomings limit the application of natural enzymes, thus stimulating the emergence and development of enzyme‐mimetic catalysts.^[^ ^11^, ^46^ ^]^

## 3 Enzyme‐Mimetic Catalysts for APEXs

**Figure 3:**
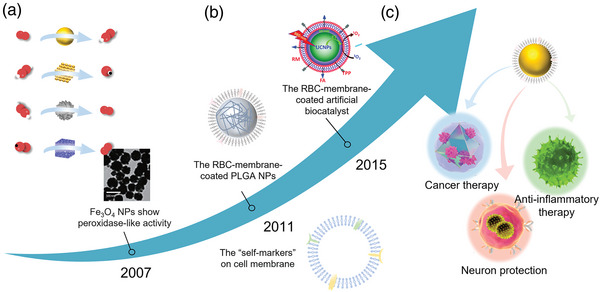
Development of enzyme‐mimetic catalysts and APEXs. a) Artificial biocatalysts have been developed that can overcome the shortcomings of natural enzyme. b) Cell membrane coating nanotechnology increases the biocompatibility and targeting ability of biocatalysts. c) APEXs for a wide range of biomedical applications. Reproduced with permission.^[^ ^48^, ^49^ ^]^ Copyright 2007, Nature Publishing Group. Copyright 2015, Royal Society of Chemistry. Fe_2_O_4_ NPs: Reproduced with permission.^[48]^ Copyright 2007, Nature Publishing Group. RBC‐membrane‐coated artificial biocatalyst: Reproduced with permission.^[49]^ Copyright 2015, Royal Society of Chemistry. Cancer therapy: Reproduced with permission.^[142]^ Copyright 2020, American Chemical Society. Anti‐inflammatory therapy: Reproduced with permission._[29]_ Copyright 2020, Elsevier. Neuron protections: Reproduced with permission.^[176]^ Copyright 2020, American Chemical Society.

Here, we focus on discussing synthesized materials with enzyme‐mimetic activities (**Figure** **3**). Enzyme‐mimetic catalysts, with the advantage of good stability, low cost, and other properties of nanomaterials, have become promising tools in the field of biomedicine for disease diagnosis and treatment.^[^ ^47^ ^]^ Four types of redox enzymes have been reported to have been mimicked by nanomaterials, including OXD, POD, CAT, and SOD, and can be used as substitutes for natural enzymes and have been developed to treat diseases.

### 3.1 OXD Mimics

**Figure 4:**
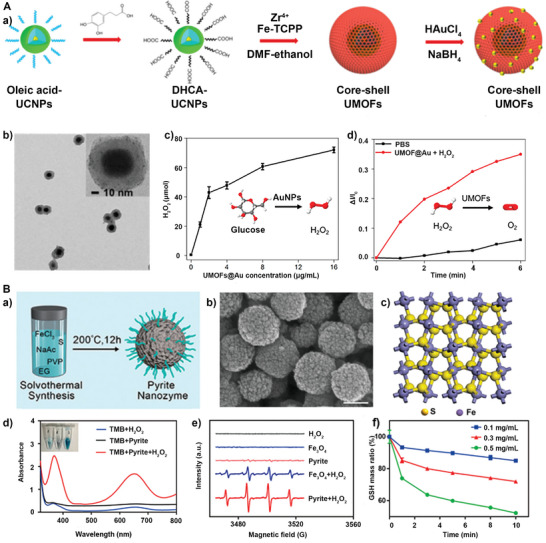
A) a) Schematic diagram of the synthesis process of UMOF@Au. b) TEM of UMOF@Au. c) H_2_O_2_, and d) O_2_ generation from UMOFs. Reproduced with permission.^[^ ^52^ ^]^ Copyright 2020, American Chemical Society. B) a) Scheme of the synthesis of pyrite nanozyme. b) SEM of pyrite nanozyme. c) Optimized bulk structure of pyrite nanozymes. d) POD activity of pyrite nanozyme in vitro. e) ESR spectra DMPO‐·OH spin adduct generated from pyrite nanozyme‐catalytic decomposition of H_2_O_2_. f) Concentration‐dependent GSH‐Ox‐mimicking activity of pyrite nanozyme. Reproduced with permission.^[^ ^54^ ^]^ Copyright 2021, American Chemical Society.

Up to now, several nanomaterials such as metal, metal oxides, and MOFs have been reported with OXD‐like activity. For example, gold nanoparticles (Au NPs) have GOx‐like activity. Among them, the metal‐based nanomaterial is one of the most potential enzyme‐mimetic catalysts, which can efficiently catalyze glucose decomposition. The “non‐naked” Au NPs (Au@BSA NPs) have been synthesized with protein as a stabilizer.^[^ ^50^ ^]^ Different from natural enzymes, the “non‐naked” Au NPs exhibit both GOx‐like and POD‐like activities at the same pH. With natural protein as the protector, Au NPs have stable GOx‐like activity. Similarly, alloys also have OXD‐like activities. The pH‐responsive graphitic nanozyme, PtCo@Graphene (PtCo@G), has also been created for the effective treatment of *Helicobacter pylori*.^[^ ^51^ ^]^ PtCo alloys exhibited OXD‐mimicking properties, and then the graphene was grown on their surface to form PtCo@G. In vitro, it was found that PtCo@G could be activated with superior OXD‐like activity under acid conditions. It has been demonstrated that metal oxides and MOF also exhibited OXD‐mimicking activities. For example, core–shell UMOF NPs were constructed using ultrasmall Au NPs as enzyme‐mimetic catalysts (**Figure** **4**Aa).^[^ ^52^ ^]^ As shown in Figure 4Ab, the ultrasmall Au NPs (≈2 nm) have evenly distributed in the MOF shell matrix. The ultrasmall An NPs still maintain their GOx activity (Figure 4Ac). And UMOF had CAT activity (Figure 4Ad), which can cascade with ultrasmall An NPs. In another example, MOF‐818 nanomaterials have been proposed, which show catechol oxidase activity characteristics without POD‐like activity.^[^ ^53^ ^]^ Because of the trinuclear copper centers that mimic the active center of natural OXD, the MOF‐818 shows highly efficient oxidase‐like activity. All these studies show that compared with natural enzymes, the catalytic activity of enzyme‐mimetic catalysts is adjustable, easy to modify, and more widely available.

### 3.2 POD Mimics

Since the Yan group first found Fe_3_O_4_ had intrinsic POD‐mimicking activity in 2007, more and more nanomaterials such as metal, metal oxide, and carbon‐based NPs have been reported to act as POD mimics. Recently, a facile one‐pot solvothermal method has been proposed via synthesizing the spherical pyrite nanozyme. (Figure 4Ba–c).^[^ ^54^ ^]^ The high affinity to H_2_O_2_ endowed pyrite nanozyme with higher catalytic efficiency than natural HRP and classical Fe_3_O_4_ nanozymes (Figure 4Bd,e). Further, they found the glutathione oxidase (GSH‐Ox) mimicking the activity of pyrite nanozymes to oxidize GSH to H_2_O_2_ and oxidized glutathione (GSSG) (Figure 4Bf). Besides the single‐metal nanostructures, it was also found that oxygen‐deficient bimetallic oxide. FeWO*_x_* nanosheets can present a higher inherent POD‐mimetic activity due to the sheet‐like structure with maximally exposed catalytic sites.

Recently, vermiculite (VMT) with an ultrathin sheet‐like structure has arisen extensive interest. The high‐quality ultrathin 2D VMT nanosheets were fabricated by a top‐down method of reflux ion exchange.^[^ ^55^ ^]^ Polyvinyl pyrrolidone (PVP) was modified on the surface of nanosheets as a stabilization agent to form VMT‐PVP with high dispersibility and POD‐mimetic activity.

Enzyme‐mimetic catalysts show enzyme‐mimetic activity which is adjustable. For example, a photoinduced enhanced CuS hollow nanocages (HNCs) nanozyme was proposed with predominant POD‐mimetic activity.^[^ ^56^ ^]^ The CuS HNC showed a superior localized surface plasmon resonance effect that can facilitate ROS production due to its hollow‐cage structure and rough surfaces. These studies indicate that some enzyme‐mimetic catalysts presented extraordinary catalytic activity and might have potential as a POD replacement.

### 3.3 CAT Mimics

Like natural enzymes, the enzyme‐mimetic catalysts can also imitate CAT to scavenge ROS.^[57]^Metal‐based nanomaterials have been reported to exhibit excellent CAT‐mimic activity, protecting normal cells from damage by cellular oxidative stress damage, which is induced by ROS. For instance, a bovine serum albumin‐iridium oxide nanoparticle (BSA‐IrO_2_ NPs) has been reported, which not only showed outstanding photothermal conversion efficiency and high X‐ray absorption capability but also had CAT‐mimicking activity.^[^ ^58^ ^]^ Further, it is demonstrated that IrO*_x_* nanozyme displays multienzyme‐mimetic activities, including POD‐ and OXD‐mimicking and pH‐dependent CAT‐mimicking activities for antitumor.^[^ ^59^ ^]^ Another work synthesized a generation 5 dendrimer/gold (G5Au) NPs that, with intrinsic CAT‐like properties, can decompose H_2_O_2_ for relieving cancer hypoxia.^[^ ^60^ ^]^

**Figure 5:**
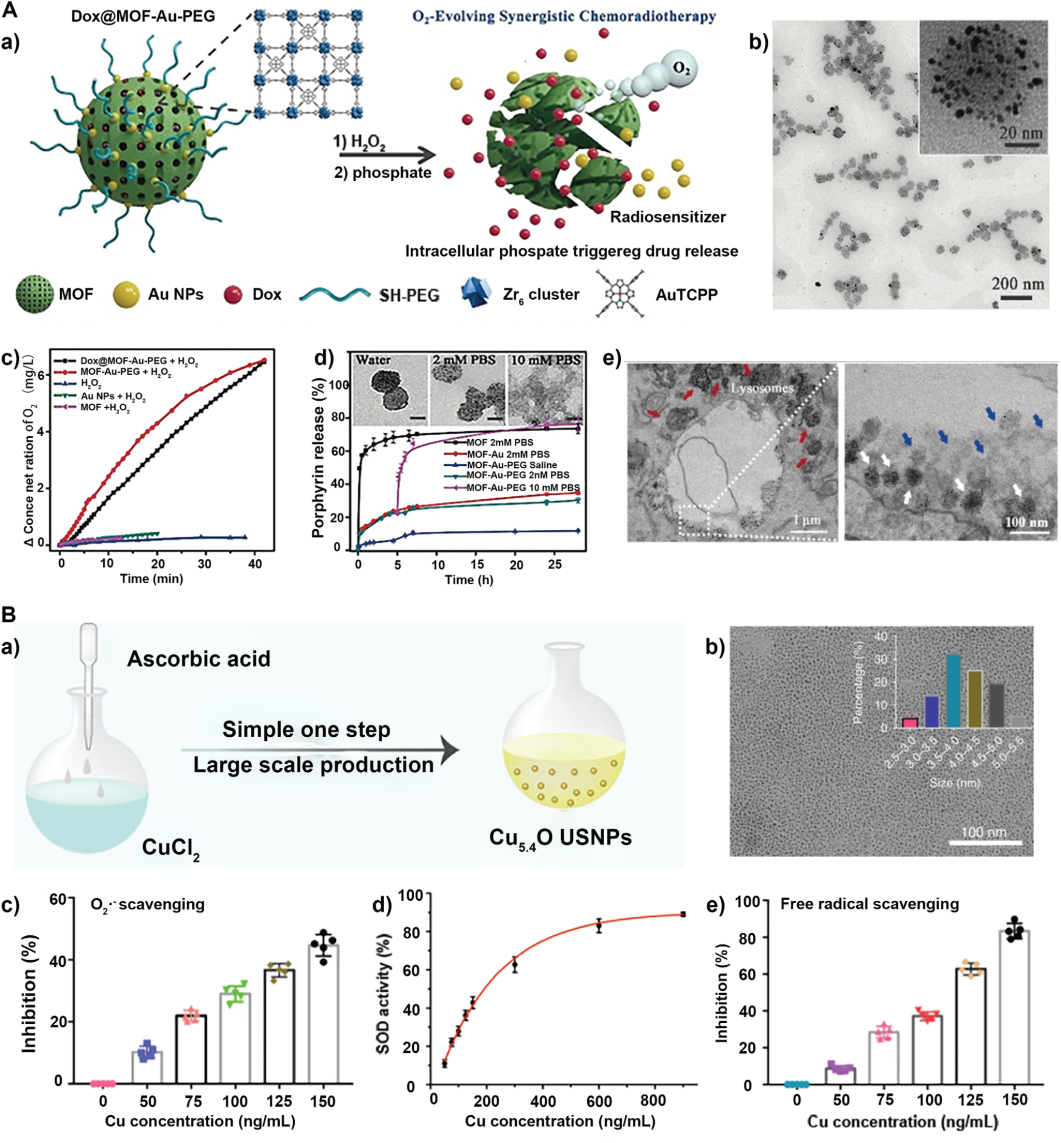
A) a) Schematic illustration of the Dox@MOF‐Au‐PEG with CAT‐like catalytic activity for O_2_‐evolving synergistic chemo‐/radiotherapy. b) TEM images of MOF‐Au NPs. c) O_2_ generation by various samples with MOF (1 mg mL^−1^) weight in the presence of H_2_O_2_. d) Release profiles and TEM images of the porphyrin ligand under different conditions. e) Bio‐TEM images of U87MG cells after treatment with MOF‐Au‐PEG. Reproduced with permission.^[^ ^62^ ^]^ Copyright 2019, Wiley‐VCH. B) a) The preparation process and b) TEM image of Cu_5.4_O USNPs. c) Superoxide anion scavenging ability, d) SOD activity, and e) antioxidant ability of Cu_5.4_O USNPs. Reproduced with permission.^[^ ^63^ ^]^ Copyright 2020, Nature Group.

In addition to metal‐based nanomaterials mentioned above, some MOF‐based nanomaterials could exhibit CAT‐like activities. For instance, a Mn_3_O_4_ nanozyme that came from Mn‐MOF and had CAT‐like properties to decompose H_2_O_2_ into O_2_ and H_2_O was fabricated.^[^ ^61^ ^]^ The obtained Mn_3_O_4_ nanoparticles exhibited high stability, biocompatibility, and a large surface area that can load photosensitizers for PDT. Similarly, a PEGylated porphyrinic MOF‐gold nanoparticles (MOF‐Au‐PEG) was fabricated as an O_2_‐evolving and drug delivery platform to realize in situ catalytic oxygenations (**Figure** **5**Aa,b).^[^ ^62^ ^]^ The MOF‐Au‐PEG showed CAT‐like activity (Figure 5Ac). On the other hand, once the concentration of PBS increased to 10 mm, the MOF skeleton collapsed and the porphyrins and Au NPs were released. (Figure 5Ad). According to the TEM analysis, MOF‐Au‐PEG NPs were fragmented inside cells (Figure 5Ae), which benefited the liberation of DOX. Thus, enzyme‐mimetic catalysts can be used not only as substitutes for natural enzymes but also as photosensitizers or nanocarriers in nanotherapeutics and biomedicines.

### 3.4 SOD Mimics

Another natural enzyme that scavenges ROS is also imitated by nanomaterials. Most reported nanomaterials had SOD‐mimicking activities along with other enzyme‐like activities. For instance, ceria nanozymes with ROS combating oxidative stress capability have been utilized in liver injury therapy, and simultaneously with CAT‐mimic activity, generating O_2_.^[^ ^64^ ^]^ Moreover, taking advantage of the SOD‐like action, a novel strategy was reported using a simple and green method to synthesize the ultrasmall Cu_5.4_O nanoparticles with multiple enzyme‐mimic properties and excellent biocompatibility (Figure 5Ba).^[^ ^63^ ^]^ TEM image showed that these Cu_5.4_O USNPs had uniform structure, with an average diameter of 3.5–4.0 nm (Figure 5Bb). The obtained ultrasmall copper‐based nanozymes simultaneously exhibit SOD‐, CAT‐, and glutathione peroxidase (GPx)‐like activities and possess good biocompatibility and high renal clearance properties (Figure 5Bc–e). In addition to metals and metal oxide nanomaterials, non‐metallic nanoparticles also have properties that mimic SOD. For example, nitrogen‐doped porous carbon nanospheres have been reported in which nitrogen was inserted into the lattice of graphite structure to perform four above enzyme‐like activities.^[^ ^65^ ^]^ All these works suggest that enzyme‐mimetic catalysts can mimic the activity of multiple enzymes simultaneously.

Although the discovery of nanozymes overcomes the shortcomings of a natural enzyme which are difficult to obtain, difficult to preserve, and high cost, compared with natural enzymes, the nanozymes exhibit lower catalytic activity and selectivity. There have been already several strategies to improve the catalytic activity of nanozymes, such as single‐atom catalysts mimicking the structure and catalytic activity of natural enzymes,^[^ ^66^, ^67^, ^68^ ^]^ ligand modification mimicking the chiral structure of natural enzymes,^[^ ^69^, ^70^ ^]^ and tailored catalytic microenvironment. With the improvement of catalytic activity nanozymes can be used to construct high‐performance APEXs for a wider range of biomedical applications.^[^ ^71^ ^]^ These natural and artificial enzymes are used for biomedicine due to their respective characteristics. However, sometimes it is difficult to obtain the desired therapeutic or diagnostic effect only using a single catalyst.

### 3.5 Nanohybrid Catalysts

To integrate the activities of natural and artificial enzymes, substantial efforts have been made to develop natural/artificial enzyme hybrids also known as nanohybrid enzymes. Nanohybrid enzymes gain their advantages by integrating the benefits of natural enzymes and nanomaterials. For example, MOF‐based catalysts can participate in cascade reactions. Many integrations of enzyme‐mimetic catalysts with natural enzymes to obtain biomimetic‐cascaded catalytic systems have been explored.^[^ ^72^ ^]^ For instance, an integrated enzyme system, Fe‐MOF‐GOx, is designed for the colorimetric detection of glucose.^[^ ^73^ ^]^ Fe‐MOF not only shows POD‐like activity,but also can immobilize GOx by covalent binding. In summary, the integrations of natural enzymes and enzyme‐mimetic catalysts can combine the advantages of both selectivity and controllable activity. In addition, metal oxide‐based nanozymes can be combined with natural enzymes to enhance each other's activity. For instance, a biomimetic nanohybrid enzyme was reported by integrating MnO_2_ and GOx, and the whole nanohybrid enzyme was named rMGB.^[^ ^74^ ^]^ MnO_2_ possesses CAT‐like activity, catalyzing the decomposition of H_2_O_2_ to produce O_2_ in solid cancers that further improves the catalytic efficiency of GOx. At the same time, the generation of gluconic acid can enhance the catalytic efficiency of MnO_2_. CAT is another natural enzyme that can be used to construct nanohybrid catalysts. For example, the encapsulation of CAT in MIL‐101‐type MOF heterostructure, in which black phosphorus quantum dots and CAT form a cascade reaction system to relieve hypoxia and achieve photodynamic‐thermal synergistic therapy.^[^ ^75^ ^]^

The main obstacle to using natural enzymes and enzyme‐mimetic catalysts in diagnosing and treating disease is the complex microenvironment in vivo. When they enter the organism, they are recognized and cleared by the endothelial reticular system, leading to a short circulation time and insufficiently targeted accumulation.

## 4 Cloaking Cell Membranes on Catalysts to Engineer Multifaceted APEXs

The cell, one of the most fundamental units of biology, performs various complex functions, including the extraordinary ability to interact with its surrounding environment.^[^ ^19^ ^]^ Cellular functions are highly associated with cell membranes. The cell membrane, a thin semipermeable membrane, is composed of lipids, proteins, and carbohydrates.^[^ ^18^ ^]^ Cell membrane‐derived vesicles have been used for the surface modification of natural enzymes and enzyme‐mimetic catalysts. Different cell membranes are made up of different constituents and have different physiological functions, so by regulating the type of cell membranes, the inner nucleus can perform the desired function (Figure 3B). We summarized the extractions and characteristics of cell membranes that have been used for surface modification of catalysts.

### 4.1 The Extraction of Cell Membranes

**Figure 6:**
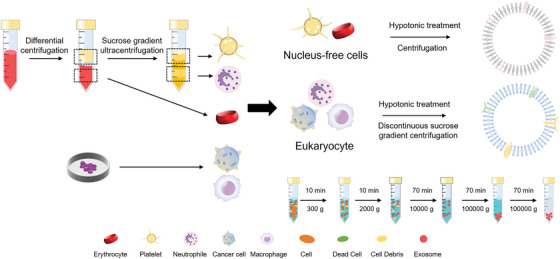
Extractions of cell membranes.

Nucleus‐free cells in humans, including red blood cells (RBCs) and platelets, are highly differentiated cells with specialized functions.^[^ ^19^, ^76^ ^]^ And the lack of a nucleus is favorable for the extraction and purification of cell membranes.^[^ ^77^, ^78^ ^]^ To obtain cell membranes from nucleus‐free cells, the cells are first isolated from whole blood through multiple centrifugation‐based methods (**Figure** **6**).^[^ ^76^ ^]^ And then treatment with hypotonic cell lysis or repeated freeze–thaw process. The soluble proteins can be removed by centrifugation to obtain the purified cell membrane fragments, which are then extruded via polycarbonate porous membranes to generate cell membrane‐derived nanovesicles (Figure 6).^[^ ^79^ ^]^

The extraction of cell membranes from eukaryotes is more complicated than nucleus‐free cells.^[^ ^76^, ^80^ ^]^ First, enough cells should be harvested from blood and tissue samples or culture dishes (Figure 6). Then the targeting cells were lysed by treating them with hypotonic lysis solutions and/or mild mechanical processes.^[^ ^81^ ^]^ The intracellular vesicles, biomacromolecules, and nuclei can be removed by discontinuous sucrose gradient centrifugation. In the final step, which is the same as the extraction of the nucleus‐free cell membrane, the resulting cell membrane vesicles were further extruded into small nanovesicles (Figure 6).^[^ ^79^ ^]^

In addition to single cell membranes, hybrid cell membranes have also been reported.^[^ ^82^ ^]^ Compared with the single cell membranes, the hybrid cell membranes can desirably integrate different biological functions from their original cells.^[^ ^83^ ^]^ To prepare hybrid cell membranes, different cell membranes are obtained separately and then fused by the extrusion method or ultrasonic method.^[^ ^84^ ^]^ Elevated‐temperature stirring has also been reported to prepare RBC–platelet hybrid membrane.^[^ ^85^ ^]^

Exosomes, recently reported natural carriers of nanoparticles, perform many unique functions, especially intercellular communication and intrinsic cellular regulations.^[^ ^86^, ^87^ ^]^ Exosomes that are secreted into body fluids by eukaryotes contain the same biological species as their source cells, including proteins, lipids, nucleic, and amino acids, which can reflect the cellular origin of exosomes.^[^ ^88^ ^]^ Exosomes can be separated from all body fluids like saliva, urine, blood, and so on.^[^ ^89^ ^]^ There are many conventional ways to isolate exosomes, such as immunoaffinity capture, size exclusion chromatography, polymer precipitation, ultracentrifugation, and density gradient centrifugation.^[^ ^90^ ^]^ Ultracentrifugation is one of the most common methods and is regarded as the gold standard (Figure 6).^[^ ^86^ ^]^ Recently, researchers have also developed a new method, microfluidics‐based exosome isolation, which can overcome the disadvantages of traditional methods, such as low yield and long time‐consumpion.^[^ ^91^ ^]^ All methods mentioned above were used to separate exosomes from apoptotic fragments.

Similar to extracellular vesicles, the bacterial outer membrane vesicles (OMVs) could also exchange information between species of bacteria.^[^ ^92^ ^]^ The isolation of bacterial membranes is difficult, owing to bacteria wrapped in cell walls that are composed of peptidoglycan. And the bacterial membrane can be replaced by the OMVs, which can be extracted from the medium through ultrafiltration. Cell lysis is unnecessary compared to the extraction of eukaryote membranes.^[^ ^76^, ^79^ ^]^

Liposomes are artificial membranes formed by phospholipids and aqueous cores that have been used as drug delivery systems. Recently, many methods have been developed to produce liposomes, including microfluidic technologies, flow focusing, pulsed jetting, double emulsion template, droplet emulsion transfer, and hydrodynamic focusing. These new methods have the advantage of adjustable size and lamellarity compared to conventional bulk methods.^[^ ^93^ ^]^

### 4.2 Nucleus‐Free Cell Membranes for APEXs

As the most abundant cells in blood circulation, RBCs live up to 120 days within the bloodstream.^[^ ^19^ ^]^ RBC membrane is a promising surface modification material for catalysts because there are many “self‐markers,” such as C8‐binding protein, homologous restriction protein, CD47, and CD59,^[^ ^94^ ^]^ which can decrease the clearance of catalysts by the reticuloendothelial system and macrophages to prolong circulation time.^[^ ^17^ ^]^ The RBC membrane is a popular natural carrier due to its ease of collection and extraction.

**Figure 7:**
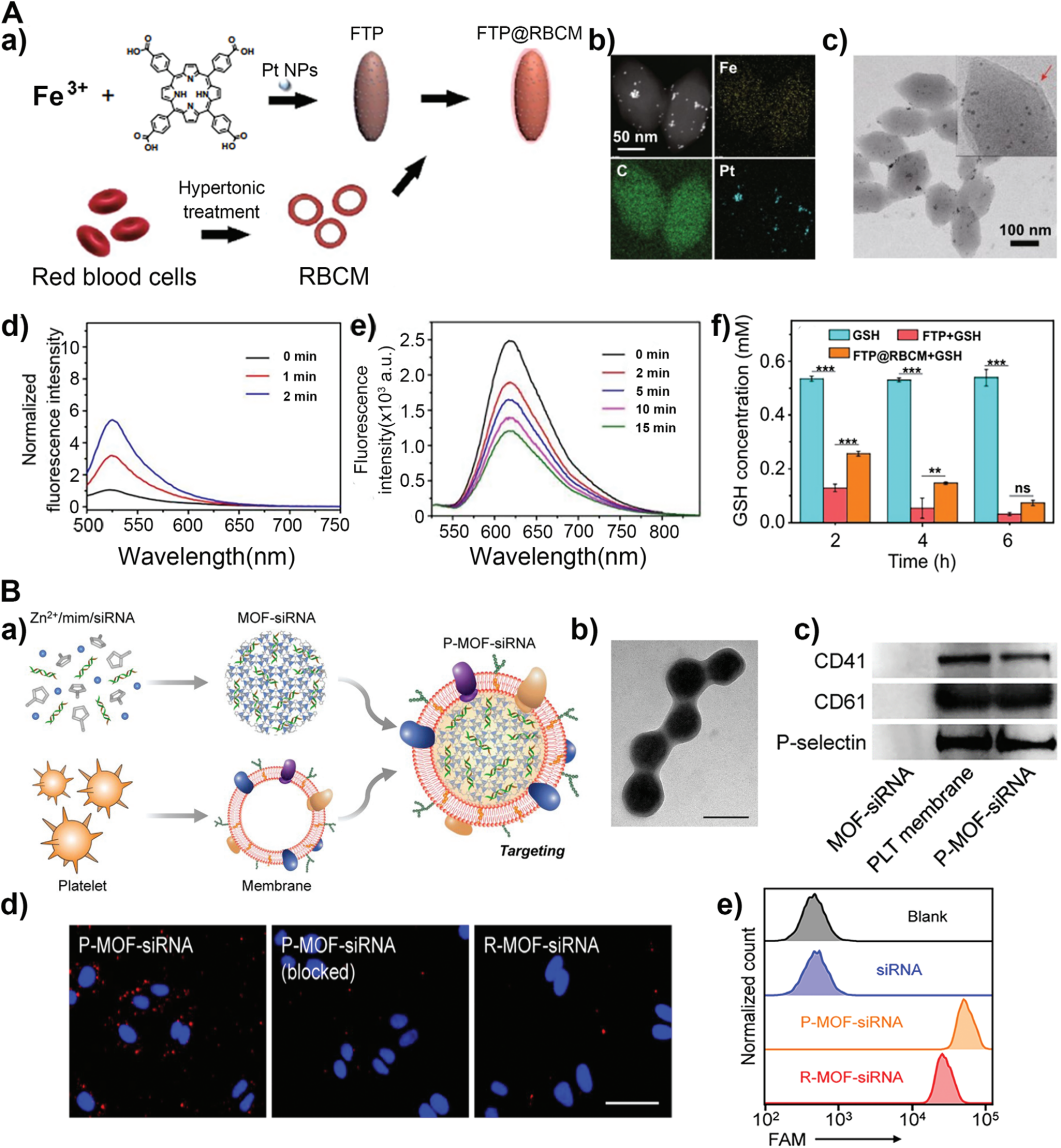
A) a) Schematic illustration of artificial RBCs (FTP@RBCM) preparation. b) TEM mapping of FTP. c) TEM image of FTP@RBCM. d) Fluorescence spectrum of SOSG incubated with FTP@RBCM. e) Fluorescence spectrum of [Ru(dpp)_3_]Cl_2_ in the presence of FTP@RBCM and H_2_O_2_. f) GSH concentration after FTP and FTP@RBCM treatment (*n* = 3). Reproduced with permission.^[^ ^96^ ^]^ Copyright 2022, Springer. B) a) Schematic diagram of the preparation of P‐MOF‐siRNA. b) TEM image of P‐MOF‐siRNA. c) Three characteristic platelet surface markers were detected by Western blot. d) Binding of fluorescently labeled P‐MOF‐siRNA, P‐MOF‐siRNA pre‐blocked with anti–P‐selectin, or R‐MOF‐siRNA to SK‐BR‐3 cells. e) The uptake of three nanoparticles in SK‐BR‐3cells. Reproduced with permission.^[^ ^98^ ^]^ Copyright 2020, AAAS.

Many types of nanoparticles camouflaged by RBC membranes can escape immunogenic clearance and prolonged blood circulation time have been reported.^[^ ^95^ ^]^ For instance, an artificial RBC (FTP@RBCM) that can produce radical storms has been developed. As shown in **Figure** **7**Aa–c, the artificial RBC was composed of Fe‐porphyrin‐based MOFswith CAT‐like and GPx‐like activities as the core, and the outer layer was the RBC membranes.^[^ ^96^ ^]^ The enzyme activity of the Fe‐protoporphyrin‐based hybrid framework (FTP) was not affected by the erythrocyte membrane (Figure 7Ad–f). In another work, nanoparticles were fabricated by wrapping DOX within hollow mesoporous Prussian blue nanoparticles, which were then further coated with an erythrocyte membrane.^[^ ^97^ ^]^ In vitro studies revealed that the nanoparticle wrapping with RBC membrane showed favorable biocompatibility and immune evading ability, which was simulated by the outer red cell membrane.

Platelets, another essential nucleus‐free cell type in humans, are released from megakaryocytes.^[^ ^19^ ^]^ Platelets are responsible for hemostasis by accumulating at vascular damage sites that lead to clot formation. Similar to RBCs, platelets also escape phagocytosis and circulate for a long time. In addition, platelets recognize and interact with circulating cancer cells, which promotes tumor metastasis.^[^ ^24^ ^]^ These functions of platelets are attributable to the surface marker proteins, including CD47, CD45, and CD59.^[^ ^99^ ^]^

A polymeric nanoparticle coated with platelet membranes was reported.^[^ ^99^ ^]^ Compared to the naked particles, nanoparticles that are cloaked with a layer of the platelet membrane lack particle‐induced complement activation and have reduced their uptake by macrophage cells. In the meantime, both in vitro and in vivo experiments validated that cloaked nanoparticles exhibit some of the properties of platelets, including enhanced binding to platelet‐adhering pathogens and selective adherence to damaged vasculatures. Benefiting from cancer‐targeting capabilities, platelets have inspired the design of a nanohybrid delivery platform. The siRNA was encapsulated within MOFs, which were then coated by platelet membranes (Figure 7Ba).^[^ ^98^ ^]^ The whole nanoplatform is named P‐MOF‐siRNA, showing cancer‐targeting properties and achieving gene silencing. The core–shell morphology confirmed by TEM and the western blotting analysis was used to verify the successful cloaking of the platelet membrane onto the MOF‐siRNA nanoparticles (Figure 7Bb,c). Fluorescence imaging revealed that platelet membrane cloaking helped to increase the affinity between nanoparticles and breast cancer cells compared with nanoparticles coated with other cell membranes (Figure 7Bd). And flow cytometry suggested that platelet membrane‐cloaked nanoparticles could be used to increase the uptake of siRNA due to the specific interactions of platelets and cancer cells (Figure 7Be). Although it is easier to extract the membranes from nucleus‐free cells, the functions of the membranes are simple and cannot meet more complex needs. Therefore, researchers have been exploring other cell membranes, such as eukaryotes, extracellular vesicles, and bacterial OMVs.

### 4.3 Cell Membranes from Eukaryocyte for APEXs

**Figure 8:**
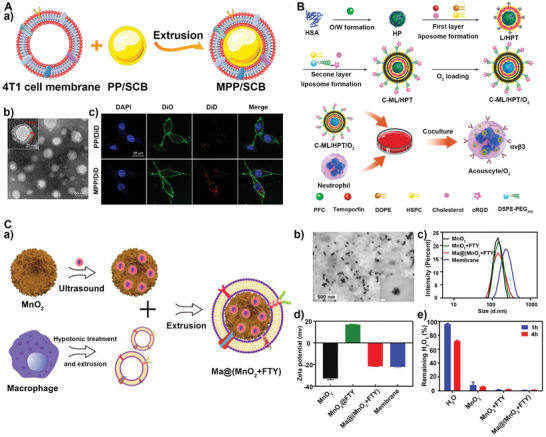
A) a) Schematic illustration of the preparation MPP/SCB. b) TEM images of MPP/SCB. c) CLSM imaging PP/DiD and MPP/DiD uptake by cells, scale bar: 20 µm. Reproduced with permission.^[^ ^103^ ^]^ Copyright 2021, American Chemical Society. B) Protocol for the preparation of Acouscyte/O_2_. Reproduced with permission.^[^ ^105^ ^]^ Copyright 2022, Wiley‐VCH. C) a) Schematic preparation of Ma@(MnO_2_ + FTY). b) TEM images of Ma@(MnO_2_ + FTY). c) Size distribution and d) Zeta potential of MnO_2_, MnO_2_ + FTY, and Ma@(MnO_2_ + FTY) nanoparticles and macrophage cell membrane vesicles. e) H_2_O_2_ scavenging behavior of MnO_2_, MnO_2_ + FTY, and Ma@(MnO_2_ + FTY). Reproduced with permission.^[^ ^106^ ^]^ Copyright 2021, Wiley‐VCH.

Eukaryocyte membranes that can be used for surface modifications of nanoparticles include cancer cell membranes, leukocyte membranes, macrophage membranes, and stem cell membranes. As mentioned above, RBC/platelet membrane‐camouflaged nanoparticles do not have a targeting ability to cancer. Cancer cell membranes have been widely used for the surface modification of nanomaterials due to their homologous adhesion to homologous cells and their natural immune escape properties.^[^ ^17^, ^81^, ^100^ ^]^ Functionalizing nanoparticles with cancer cell membranes can make the resulting particles possess some unique properties closely resembling that of the source cells.^[^ ^101^, ^102^ ^]^ For example, inspired by the ability of 4T1 tumor cells to penetrate the blood–brain barrier (BBB), He and co‐workers reported a biomimetic nanocarrier (MPP/SCB) last year, which is based on the 4T1 cell membrane.^[^ ^103^ ^]^ MPP/SCB is composed of the outer 4T1 cell membrane and the inner antioxidant succinobucol (SCB), which can reduce cerebral ischemia/reperfusion injury (**Figure** **8**Aa). The TEM measurements showed that MPP/SCB was homogeneous spherical particles with an obvious core–shell structure (Figure 8Ab). The nanovesicles were labeled with a far‐red plasma membrane fluorescent probe (DiD), and confocal laser scanning microscopy (CLSM) showed that MPP/DiD was more easily internalized by PC12 cells (Figure 8Ac). In another case, dexamethasone (DXM)‐loaded IFN‐*γ*‐treated major histocompatibility complex‐deficient cancer membrane‐cloaked nanoparticles (IM‐MNPs/DXM) were fabricated to exploit the immunosuppressive ability of tumor cells for the treatment of systemic lupus erythematosus (SLE).^[^ ^104^ ^]^

Besides cancer cell membranes, many other eukaryote membrane types have been used to decorate nanomaterials, such as neutrophils, the most abundant type of leukocytes in the human blood, are the first line of defense of the immune system and play an essential role in innate immunity.^[^ ^107^ ^]^ Neutrophils can respond to tissue damage or infections by initiating inflammation. In the inflammation process, neutrophils are activated and subsequently migrate to invading pathogens.^[^ ^105^, ^108^ ^]^ Neutrophils can also drive to the tumor site due to chemokine recruitment. For instance, a sonosensitizer cell, called Acouscyte/O_2_, is made of customized liposomes with neutrophils.^[^ ^105^ ^]^ In vitro/in vivo experiments proved that Acouscyte/O_2_ can be selectively accumulated in the tumor, which was attributed to the adhesion molecules on the neutrophil membranes to realize the integration of diagnosis and treatment. As shown in Figure 8B, Acouscyte/O_2_ consisted of liposomes encapsulated with oxygen carried perfluorocarbon and temoporfin and live neutrophils. Based on the suppression of pro‐arthritogenic factors and cartilage penetration, the neutrophil membrane‐camouflaged nanomaterials are also attractive for inhibiting synovial inflammation.^[^ ^109^ ^]^

Macrophages are also inflammatory‐related cells that are essential for the removal of dead cells and derbies, control and clearance of infections, and promoting wound healing and tissue repair.^[^ ^110^ ^]^ Proteomic analysis by some investigators found that macrophage camouflage membrane PLGA exhibited inflammatory targeting ability in vitro/in vivo due to CD44 and macrophage‐1 antigen (Mac‐1) expression on the macrophage surface.^[^ ^111^ ^]^ Interestingly, the phenotype and function of macrophages change with the surrounding microenvironment.^[^ ^112^ ^]^ Similarly, macrophage membrane‐wrapped cores have the properties of macrophages. For example, the characteristic that macrophage‐membrane proteins can be recognized by adhesion molecules can enable the accumulation of biomimetic nanoparticles modified by macrophage membranes in the brain to treat ischemic stroke (Figure 8Ca).^[^ ^106^ ^]^ The capsule‐nucleus structure and the membrane Zeta potential similar to the macrophage membrane showed that the macrophage membrane was successfully encapsulated on the surface of MnO_2_ nanospheres (Figure 8Cb–d). Cloaking of the macrophage cell membrane did not affect the ROS scavenging ability of MnO_2_ (Figure 8Ce).

In addition to those mentioned above, other eukaryote membranes can also be used to construct APEXs, such as bone marrow stromal membranes being used to develop a biomimetic nanocomposite to eliminate leukemia cells in the bone marrow.^[^ ^113^ ^]^ Mesenchymal stem cells (MSCs) are derived from bone marrow, umbilical cord, and adipose tissue, which were commonly utilized in clinical disease treatment. Using the hypoimmunogenic and active tumor‐targeting MSC membrane rather than the whole MSCs can better eliminate the immune and pathological reactions caused by the whole cells.^[^ ^114^ ^]^ A *β*‐NaYF4:Yb^3+^,Er^3+^ upconversion nanoparticle was first encapsulated in mesoporous silica and then fused with MSC membranes to form a biomimetic tumor PDT platform, which inherits the tumor‐targeting properties of MSCs, exhibits remarkable accumulation at the tumor site, and show higher tumor inhibition efficacy.^[^ ^115^ ^]^ Another siRNA delivery and photothermal therapy nanoplatform based on Fe_3_O_4_@PDA–siRNA@MSCs nanoparticles could inhibit the expression of endogenous Plk1 gene and lead to obvious apoptosis in prostate cancer cells.^[^ ^116^ ^]^  Eukaryocyte membranes have more physiological properties than nucleus‐free cell membranes, but their limited sources and complex extraction processes limit their mass production.

### 4.4 Cell Membranes from Extracellular Vesicles and Bacterial OMVs for APEXs

Inspired by their structural stability, crossing vascular endothelium, homologous targeting capability, and wide sources, ^[^ ^117^ ^]^ exosomes have been widely employed as natural delivery platforms.^[^ ^118^, ^119^ ^]^ For example, biomimetic nanoparticles that combine the advantage of exosomes and nanomaterials were developed.^[^ ^120^ ^]^ The DOX@E‐PSiNPs were prepared by incubating cancer cells with DOX‐loaded PSiNPs (DOX@PSiNPs) for targeted cancer chemotherapy. After intravenous injection, the nanocarriers show enhanced cancer accumulation and penetration, improving the efficiency of cancer treatment. Another example, on cloaking with extracellular vesicles, nanoparticles can escape from the phagocytosis of mononuclear phagocytes.^[^ ^117^ ^]^ A microfluidic sonication approach to composite exosome membrane‐cloaked nanoparticles was explored in a one‐step method. Exosomes from cancer cells show CD47 on their membranes.^[^ ^121^ ^]^ Therefore, the engineered EM‐PLGA nanoparticles showed increased cancer‐targeting ability and decreased uptake by mononuclear phagocytes compared to lipid‐PLGA nanoparticles and CCM‐PLGA nanoparticles.

**Figure 9:**
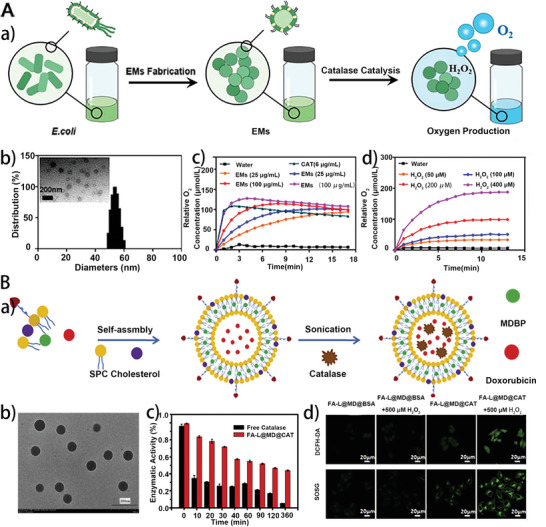
A) a) The preparation scheme of EMs and their catalytic oxygen generation. b) TEM image and size distribution of EMs. c) Different concentrations of EMs catalyze H_2_O_2_ to produce oxygen. d) EMs catalyzes the production of oxygen from different concentrations of H_2_O_2_. Reproduced with permission.^[^ ^124^ ^]^ Copyright 2021, American Chemical Society. B) a) Fabrication and b) TEM image of FA‐L@MD@CAT. c) Comparison of enzymatic activities between FA‐L@MD@CAT and free CAT. d) Singlet oxygen production under different treatments was detected by 2ʹ,7ʹ‐dichlorodihydrofluorescein diacetate (DCFH‐DA) and SOSG under hypoxia (2%) conditions. Reproduced with permission.^[^ ^32^ ^]^ Copyright 2020, Elsevier Ltd.

OMVs are spherical proteolipids budding from the outer membranes of Gram‐negative bacteria and have a similar composition as bacterial membranes. Therefore, OMVs also possess homotypic targeting functions.^[^ ^122^ ^]^ Enriched with virulence factors, proteins, and toxins, OMVs can interact with the hosts to promote their internalization within the host.^[^ ^123^ ^]^ OMVs from pathogenic bacteria has been widely used as immunostimulants and vaccine platforms because of their immunomodulatory activities.^[^ ^92^ ^]^ For instance, the CAT‐containing *Escherichia. coli* membrane vesicles (EMs) were developed to alleviate hypoxia and enhance radiation therapy (RT).^[^ ^124^ ^]^ CAT containing EMs with a diameter of 60 nm were obtained by strong shear force (**Figure** 9Aa,b). Next, the researchers tested CAT activity by measuring the amount of oxygen produced by EMs incubated with H_2_O_2_. The CAT activity of EMs was higher than that of free CAT (Figure 9Ac,d). Moreover, it has been reported that OMVs can be used for targeting photothermal treatment applications.^[^ ^125^ ^]^ OMVs from transgenic *E. coli* were modified with a photosensitizer, indocyanine green (ICG), and *α*_v_ *β*_3_ integrin targeting ligand to form I‐P‐OMVs, which could be used to treat melanoma. Because of the nanosize of OMVs, I‐P‐OMVs can penetrate the stratum corneum. And I‐P‐OMVs with NIR irradiation exhibit significant therapeutic effects and delay the release and metastasis.

However, as a natural membrane, cell membranes cannot be produced in large quantities due to their limited source and the complexity of extraction. In addition, cell membrane‐based nanoparticles can only be self‐implanted, limiting their applications. Researchers have investigated liposome membranes as cloakings for decorating their nanomaterials.

### 4.5 Phospholipid Membranes for APEXs

As a novel drug delivery system, liposomes show distinctive characteristics, such as no immunogenicity, biocompatibility, and modifications on their surface.^[^ ^126^ ^]^ Nanoparticles wrapped with phospholipid membranes can enhance the permeation and retention effect, and the nanoparticles can accumulate in desired lesion sites.^[^ ^127^ ^]^ In one example, exosomes‐liposomes hybrid nanoparticles (gETL NPs) to load anticancer drugs were developed, which were designed to overcome the delivery limitation in the treatment of metastatic peritoneal carcinoma (mPC).^[^ ^128^ ^]^ The gETL NPs can accumulate preferentially in mPC and efficiently release cargo utilized in HIPEC at the hypothermia condition. In another example, phospholipid membranes were coated on the surface of photosensitizer, CAT, and DOX mixtures to obtain FA‐L@MD@CAT, which can improve intratumoral O_2_ levels via catalyzing the decomposition of endogenous H_2_O_2_, facilitating anticancer immunities and increasing chemo‐PDT (Figure 9Ba,b).^[^ ^32^ ^]^ FA‐L@MD@CAT could protect enzymatic activity for a long time and more easily taken up by cells to produce ROS than free CAT (Figure 9Bc,d)

To improve the stability of liposome drug delivery, nanobowl‐supported liposomal DOX (DOX@NbLipo) was further designed.^[^ ^129^ ^]^ The structural support engineered in liposome improved drug loading, prolonged time in blood circulation, prevented drug leakage, and did not affect the release of DOX. In vitro and in vivo experiments with DOX encapsulated with liposomes and NbLipo, respectively, also demonstrated the above advantages.

**Figure 10:**
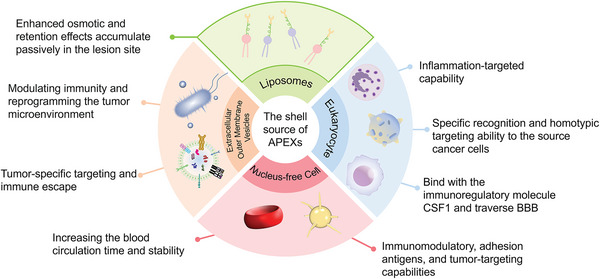
Sources and advantages of membranes for APEXs.

Different cell membranes have different extraction methods, functions, advantages, and disadvantages (**Figure** **10**). On the one hand, cell membrane from endogenous cells is much safer and friendlier compared with artificial membrane. On the other hand, the sources of artificial membranes are more extensive and more accessible to mass production than natural membranes. But both natural and artificial are desired surface modification materials of catalysts, which provide a new idea for the clinical applications of catalysts.

Due to their semipermeable membrane shell, APEXs have immune evasion and superior targeting capacity. Once in the body, the nucleus of APEXs can balance ROS by producing or scavenging ROS. Increasing ROS levels can cause lipid peroxidation and oxidative damage of biomacromolecules, thus killing tumor cells. On the contrary, the removal of ROS can reduce the damage of ROS to the body's normal tissues and thus reduce the inflammatory response.

## 5 Biomedical Applications of Engineered Multifaceted APEXs

**Figure 11:**
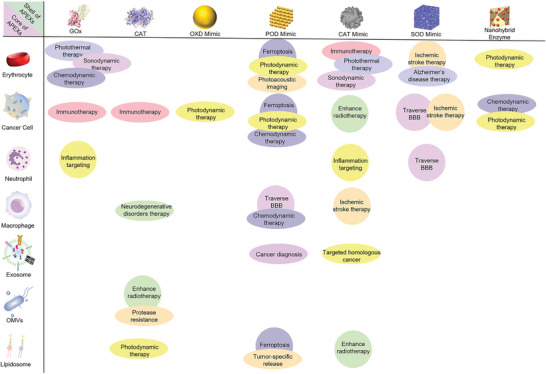
Summary of the shell and core of APEXs and their applications in biomedicine.

In the above sections, the design and fabrication of multifaceted APEXs based on cell membrane‐cloaked catalysts have been systematically discussed. The limitations of catalysts, such as passive immune clearance and nonspecific tissue accumulation, can be tackled by encapsulating cell membranes. Simultaneously, catalysts can be endowed with the biological functions of the cell membranes. Recently, multifaceted APEXs have been used to diagnose and treat diseases, including cancer, inflammation, stroke, neurodegenerative disorders, and so on (Figure 3C). This section summarizes the main applications and characteristics of APEXs (**Figure** **11**). The cell membrane, the type of catalyst core, the mimetic enzymes, and their application are summarized in **Table** **1**.

**Table 1:** Type of cell membranes used for cloaking catalysts (catalyst cores and mimetic enzymes) and corresponding biomedical applications

Cell membranes	Catalysts	Mimetic enzymes	Applications	Refs.
Erythrocyte membranes	PML	CAT	Anticancer	[130]
	FeTCPP/Fe_2_O_3_ MOF	POD	Anticancer	[131]
	Mn_3_O_4_	SOD	Ischemic stroke therapy	[132]
	GQDzyme	POD	PAI	[133]
	QD@P	CAT	Anticancer	[134]
	GOx‐Fe^0^	OXD/POD	Anticancer	[135]
	Hb@GOx	OXD	Anticancer	[136]
	Cu_x_O	CAT/SOD/GP*_X_*	Alzheimer's disease therapy	[137]
	DOX/MnTPPS	CAT	Anticancer	[138]
	TGZ@eM	OXD	Anticancer	[7]
	PBMn‐DOX	CAT	Anticancer	[139]
	MGB	CAT/OXD	Anticancer	[74]
	PCF GOx	OXD	Anticancer	[140]
	FeN200@GOx	OXD/POD	Anticancer	[141]
	NMIL‐100@GOx	OXD/POD	Anticancer	[142]
Cancer cell membranes	CMSNs	CAT/POD	Anticancer	[143]
	MC	CAT	Anticancer	[144]
	Catalase@GOx@PCN‐224	OXD/CAT	Anticancer	[145]
	CMSN‐GOx	OXD	Anticancer	[146]
	AAO@HFe–TA	OXD/POD	Anticancer	[147]
	Au@Rh‐ICG	CAT	Anticancer	[148]
	HMPB@GOx	SOD/CAT/POD/OXD	Anticancer	[149]
	ZCD	CAT	Anticancer	[150]
	GOx@ZIF‐8@BDOX	OXD	Anticancer	[151]
	MPBzyme	SOD/CAT	Ischemic stroke therapy	[152]
	PFTT	POD/CAT	Anticancer	[153]
	HLPC	OXD	Anticancer	[154]
Neutrophil membranes	GC	OXD	Anticancer/antibacterial	[155]
	MnO_2_/Fla	CAT	Anti‐inflammation	[156]
	CSPQ	SOD/POD/CAT	Treat Parkinson's disease therapy	[157]
Macrophage membranes	Catalase	CAT	Neurodegenerative disorders therapy	[158]
	PM@P NGs	POD	Anticancer	[159]
	MnO_2_ + FTY	CAT	Ischemic stroke therapy	[106]
Macrophage‐liposome hybrid membranes	nano‐Pt/VP	CAT	Anticancer	[28]
Exosome membranes	PMA/Fe‐HSA@DOX	CAT	Anticancer	[160]
	Au	POD	Cancer diagnosis	[161]
*E. coli* membrane vesicles	Catalase	CAT	Anticancer	[124]
Phospholipid membranes	CaFe@DMSN	POD	Anticancer	[162]
	CAT	CAT	Anticancer	[163]
	MD@CAT	CAT	Anticancer	[32]
	CAT@Pt	CAT	Anticancer	[164]
	Catalase	CAT	Anticancer	[165]

### 5.1 Cancer Therapies

**Figure 12:**
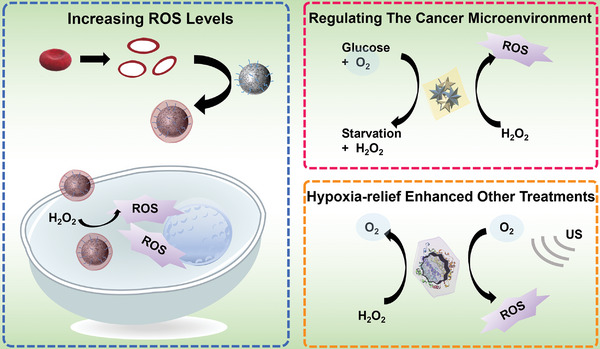
Effective pathways of APEXs in facilitating cancer treatments.

Cancer remains threatens the lives of most people in the world, despite the combined efforts of scientists and clinicians.^[^ ^16^ ^]^ It is well known that conventional cancer therapies include surgery, radiotherapy, and chemotherapy. However, because of their side effects and high recurrence rate, many efforts have been made to explore novel strategies for cancer therapy.^[^ ^166^ ^]^ Compared to bare catalysts, cell membrane‐cloaked catalysts, or APEXs, have attracted extensive interest due to their highly biocompatible, cancer‐targeting ability, especially in cancer therapy. As shown in **Figure** **12**, APEXs kill cancer cells in several strategies. The first is to kill cancer cells by increasing ROS levels. The second is regulating the tumor microenvironment (TME), such as consuming glucose for starvation therapy. The third is to enhance other therapies by increasing oxygen levels. In addition, camouflaging catalysts with cell membranes can enhance the circulation time and cancer‐targeted activities of catalysts.

#### 5.1.1 Cancer Therapies by Increasing ROS Levels

Cell membrane‐camouflaged catalysts with POD‐like activities can perform Fenton reaction, which significantly increased the concentrations of ROS to kill cancer cells directly by decomposing intratumoral overexpressed H_2_O_2_ in the TME.^[^ ^167^ ^]^ Furthermore, ROS can be produced through GOx‐based nanohybrid enzymes,^[^ ^135^, ^141^, ^149^ ^]^ in which GOx can catalyze glucose into gluconic acid. And Fe‐based enzyme‐mimetic catalysts can ionize into Fe^2+^ in acidic conditions to catalyze H_2_O_2_ to produce •OH, killing cancer cells. The outer membrane of APEXs not only prolongs blood circulation but also increases aggregation at tumor sites. For example, artificial natural killer (NK) cells have been synthesized recently with ROS‐producing ability through emulsifying the mixture of perfluorohexane, GOx, and RBC membrane.^[^ ^140^ ^]^ The obtained NK cells can consume glucose and produce H_2_O_2_ to kill cancer cells. More importantly, the artificial NK cells could generate inflammation to recruit immune cells. In a similar work, CaO_2_ and Fe_3_O_4_ NPs were loaded onto dendritic mesoporous silica nanoparticles (MSN) and wrapped with a pH‐responsive membrane.^[^ ^162^ ^]^ Through a cascade reaction, the formulation can realize the synergism of immunomodulation/immunotherapy and ferroptosis for cancer therapy.

**Figure 13:**
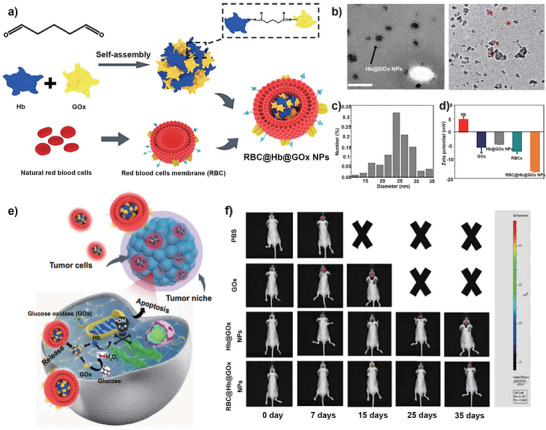
a) The preparation process of RBC@Hb@GOx NPs. b) TEM image, c) size distribution, and d) Zeta potential of RBC@Hb@GOx NPs. e) Scheme ROS production by RBC@Hb@GOx NPs for GBM treatment. f) Representative bioluminescent images of tumor‐bearing mice with U87MG in different groups for 35 days. Reproduced with permission.^[^ ^136^ ^]^ Copyright 2020, Springer.

Similarly, in another work, the protein superstructure (Hb@GOx NPs) was coated with the RBC membrane to create a novel chemodynamic therapy (CDT) nanoagent.^[^ ^136^ ^]^ Unlike loading into nanocarriers, they formulated Hb@GOx NPs through assembly and crosslinking techniques. Then the Hb@GOx NP is further camouflaged by the RBC membrane to facilitate the nanoparticle delivery across the BBB and blood–brain cancer barrier (BBTB) for glioblastoma therapy (**Figure** **13**a). TEM showed that the morphology of the Hb@GOx and RBC@Hb@GOx was uniform, and the average particle size increased to 51.55 nm after being camouflaged by the RBC membrane (Figure 13b,c). In this work, the hemoglobin (Hb) containing Fe^2+^ cofactors can act as Fenton‐like catalysts. At the same time, GOx catalyzes glucose to produce gluconic acid and H_2_O_2_, boosting the Fenton reactions to produce hydroxyl radicals that kill cancer cells directly and show powerful antitumor efficacy (Figure 13e,f).

#### 5.1.2 Cancer Therapies by Regulating Cancer Microenvironments

**Figure 14:**
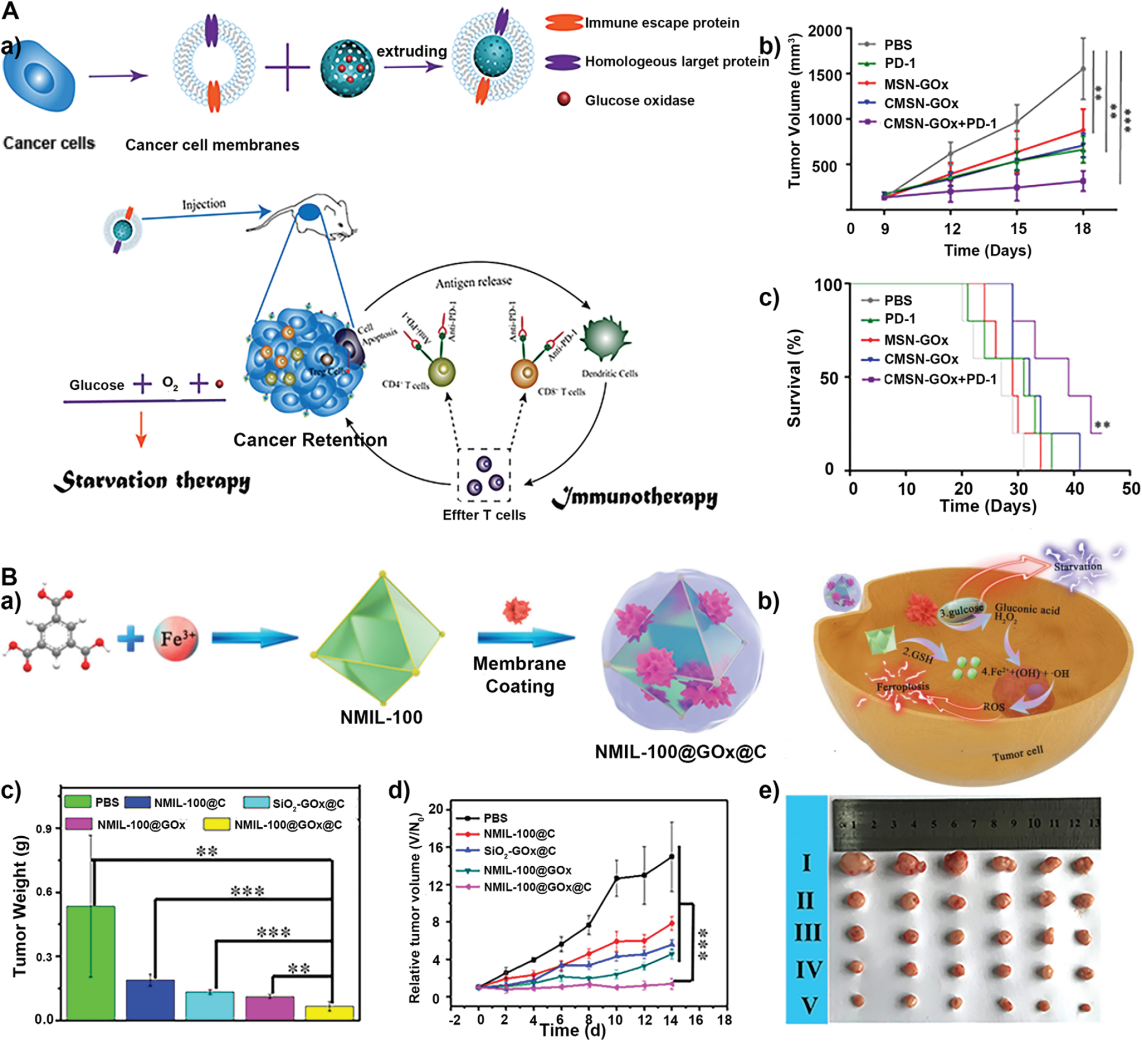
A) a) Schematic diagram of CMSN‐GOx synthesis and induced anticancer immune response and enhanced immune checkpoint‐blocking therapy. b) Tumor volume after different treatments. c) Survival curves of mice after different treatments. Reproduced with permission.^[^ ^146^ ^]^ Copyright 2019, American Chemical Society. B) a) The preparation process of NMIL‐100@GOx@C. b) The cascade processes of cancer therapy. c) Mean tumor weights and d) growth curves after different treatments. e) Picture of tumors dissected on the 14th day after different treatments (I: PBS, II: NMIL‐100@C, III: SiO_2_‐GOx@C, IV: NMIL‐100@GOx, V: NMIL‐100@GOx@C). Reproduced with permission.^[^ ^142^ ^]^ Copyright 2020, American Chemical Society.

The local microenvironment of a tumor plays an increasingly important role in the development of tumors.^[^ ^168^ ^]^ APEXs can inhibit cancer cell growth by regulating the TME. Glucose‐reducing starvation therapy can suppress the growth of cancer, and GOx‐mediated starvation therapy is often combined with other therapies. A series of cascade nanoreactors using cell membranes as decorating moieties have been established to realize immune escape and homotypic targeting behaviors. In their studies, they coated various cell membranes with the complexes of GOx and cancer drugs or nanoparticles for synergetic starvation therapy and PDT,^[^ ^145^ ^]^ chemotherapy,^[^ ^151^ ^]^ CDT,^[^ ^142^ ^]^ and immunotherapy.^[^ ^146^ ^]^ As a representative example, Wei et al. proposed a method to combine starvation therapy and immune checkpoint‐blocking therapy for targeted cancer treatment by wrapping MSN loaded with GOx in the cancer cell membranes (**Figure** **14**A).^[^ ^146^ ^]^ They have proved that the cancer membrane enables the CMSN‐GOx to target cancer and enrich cancer tissues. The integrated CMSN‐GOx complex could stimulate an antitumor immune response by inducing dendritic cell maturity and ablating tumors in vivo. Further in vivo tests also showed the better anticancer effect of CMSN‐GOx combined with anti‐PD‐1. Similarly, a biomimetic nanoreactor was reported via wrapping prodrug tirapazamine (TPZ) and GOx in an RBC membrane‐cloaked zeolitic imidazolate frameworks‐8 (ZIF‐8).^[^ ^7^ ^]^ The RBC membrane‐cloaked nanoreactor with the ability of immunity escape sustains the catalytic activity of GOx and aggravates the hypoxic microenvironment to activate the prodrug TPZ for colon cancer therapy.

Based on the fact that GOx's ability to catalyze glucose generation of H_2_O_2_, a cancer cell membrane‐cloaked cascade nanoreactor (NMIL‐100@GOx@C) was developed, which is based on GOx and Fe‐MOF starvation therapy and ferroptosis anticancer therapy (Figure 14Ba).^[^ ^142^ ^]^ The cancer cell membrane endowed the nanoreactor with homologous targeting ability to tumor and immune escaping ability. Furthermore, the high concentration of GSH in cancer sites could reduce Fe^3+^ in MOF to Fe^2+^. With the existence of Fe^2+^ and H_2_O_2_, which are generated from glucose catalysis by GOx, highly toxic •OH radicals were produced via the Fenton‐like reaction to perform ferroptosis therapy (Figure 14Bb). Notably, the in vivo anticancer activity of these nanoreactors with the ferroptosis starvation treatment group inhibited the tumor growth most efficiently and did not regrow or recurrence occurred even in the late period of treatment, indicating that synergistic therapy improved the anticancer efficacy (Figure 14Bc–e).

Compared with normal cells, cancer cells have a more active glycolytic reaction resulting in the overexpression of lactic acid, which contributes to immune suppression, cancer invasion, and metastasis.^[^ ^169^ ^]^ Therefore, targeting LA metabolism is a promising tumor treatment strategy. A study has designed an intra/extracellular lactic acid exhaustion cascade catalytic nanosystem in combination with anti‐programmed cell death ligand 1 (PD‐L1) for anticancer therapy.^[^ ^130^ ^]^ Hollow MnO_2_ loading with glycolysis inhibitor and lactate oxidase (LOx) was further coated by a layer of the erythrocyte membrane. LOx can remove lactic acid to enhance anticancer immunotherapy. At the same time, the coating of the erythrocyte membrane significantly hindered the endocytosis of macrophages. In another example, utilizing LOx converts LA into pyruvic acid and H_2_O_2_ to support a form of chemiexcited PDT.^[^ ^154^ ^]^ The self‐assembled NPs were generated by the self‐assembly of Hb, CPPO‐Ce6, and LOx. The NPs are then encapsulated with cell membranes from U251 glioma cells to form M@HLPC. M@HLPC could cross the BBB and realize metabolism‐based synergistic therapy for glioblastoma multiforme.

In addition to reducing glucose and lactate, reducing amino acids, the fundamental building blocks of proteins in cells, also inhibit cancer growth. An amino acid oxidase (AAO) delivery system (M@AAO@HFe‐TA) was constructed by incorporating AAO in hollow Fe^3+^/tannic acid (HFe–TA) nanocapsules, which are coated with the cancer cell membrane.^[^ ^147^ ^]^ Thanks to the encapsulation of cancer cell membranes, AAO could effectively accumulate at the cancer site. AAO consumed the amino acids, and HFe–TA mediated the Fenton reaction to produce ·OH. Due to the combined effects, the system activated Bcl‐2/Bax/Cyt C/caspase 3 mitochondrial apoptotic pathway to achieve cancer inhibition.

#### 5.1.3 Cancer Therapies by Enhancing Hypoxia Relief

Hypoxia (O_2_ partial pressure of 15 mmHg) is one of the characteristics of TME, which can promote tumor proliferation and affect the treatment effect.^[^ ^170^ ^]^ Plenty of APEXs have been explored to alleviate hypoxia via membrane encapsulation of CAT or its analogs and other nuclear components. As the hypoxia eases, other treatments can be enhanced, including PDT,^[^ ^32^, ^131^, ^143^ ^]^ SDT,^[^ ^134^, ^138^ ^]^ chemotherapy,^[^ ^139^, ^165^ ^]^ and RT.^[^ ^124^, ^163^ ^]^

There have been many reports about cancer therapy that is treated with APEXs‐mediated PDT. For example, the cloaking of cancer cell membranes on the surface of Au@Rh nanostructures loaded with photosensitizer ICG was demonstrated to form Au@Rh‐ICG‐CM.^[^ ^148^ ^]^ The Au@Rh core–shell nanostructure exhibiting CAT‐like activity catalyzes endogenous H_2_O_2_ to generate oxygen. After cloaking, the cancer cell membrane endows Au@Rh nanostructures with cancer‐targeting capability, allowing Au@Rh‐ICG‐CM to accumulate efficiently in the cancer site for improved PDT specificity. In another example, RBC membrane‐cloaked nanohybrid enzyme, which integrates nanozyme MnO_2_ with natural enzyme GOx and BSA‐Chlorine e6 (BSA‐Ce6), was developed.^[^ ^74^ ^]^ The obtained biomimetic hybrid nanoparticle (named rMGB) NPs exhibited the capability for PDT of cancer under irradiation. Aiming at improving the efficiency of cancer treatment, the rMGB could catalyze endogenous H_2_O_2_ decomposition to produce O_2_, facilitating GOx‐mediated starvation therapy and enhancing PDT.

The artificial membrane is another typical membrane for APEXs. A biomimetic liposomal nanoplatinum was fabricated by loading platinum nanoparticles (nano‐Pt) into liposomes and photosensitizer verteporfin (VP) into the lipid bilayer.^[^ ^28^ ^]^ And then, murine macrophage cell membranes were hybridized into the liposomal membrane to give the liposomal system targeting features. Nano‐Pt not only enhances PDT because of its CAT‐like properties but also can be used for chemotherapy due to cytotoxic activity. The liposomal system would effectively be home to the cancer site after intravenous injection, where nano‐Pt catalyzes H_2_O_2_ to provide oxygen for significantly increased PDT efficacy. Moreover, ROS generated by PDT is not only applied for killing cancer cells but also applied to trigger nano‐Pt release for chemotherapy.

SDT is another typical oxygen‐dependent therapy, which can also be enhanced by improving oxygen levels in the cancer microenvironment. For example, biomimetic nanoparticles (QD@P) Rs were designed via cloaking Ag_2_S quantum dots in RBC membrane vesicles.^[^ ^134^ ^]^ In this work, Ag_2_S quantum dots were first used as a sonosensitizer to produce ROS upon ultrasonic irradiation. CAT in RBCs stimulated the encapsulation of the nanomaterials in RBC membranes to catalyze the decomposition of endogenous H_2_O_2_, which can be increased by oral administration of the anticancer drug phenethyl isothiocyanate (PEITC). The SDT effect on cancer increased significantly with the oxygen levels increasing. In another case, researchers also utilized the natural CAT in RBCs to develop an oxygen self‐production RBC carrier system to enhance SDT.^[^ ^138^ ^]^ Both DOX and sonosensitizer, Mn‐TPPS, were encapsulated inside RBCs to obtain DOX/MnTPPS@RBCs. Due to CAT contained in RBCs, the SDT efficacy of DOX/Mn‐TPPS@RBCs was improved.

It has been reported that the hypoxic TME can be relieved by APEXs to the cancer site to enhance the therapeutic efficacy of chemotherapy.^[^ ^171^ ^]^ A nanocarrier named Prussian blue/manganese dioxide (PBMn)−DOX@RBC has been constructed.^[^ ^139^ ^]^ The existence of the RBC membrane could reduce the systemic toxicity of MnO_2_ and GOx and achieve the long circulation effect. The PBMn nanoparticles were used as a catalyzer for H_2_O_2_ activation to relieve tumor hypoxia and enhance cancer chemotherapy. In another example, a multifunctional biomimetic core–shell nanoplatform (mZCD) was reported, which could improve synergetic chemotherapy and immunotherapy.^[^ ^150^ ^]^ Herein, the nanoplatform is composed of the CAT and DOX‐loaded pH‐sensitive ZIF‐8 as the inner cores and murine melanoma cell membranes as the outer shells. Due to the immune escape ability and homologous targeting ability of cancer cell membrane, it would accumulate in cancer tissues. And CAT would decompose H_2_O_2_ to produce O_2_, leading to reduce hypoxia‐inducible factor‐1*α* (HIF‐1*α*), which would further downregulate PD‐L1 to strengthen the immune response. Therefore, mZCD exhibited the most significant cancer‐targeting and cancer‐killing abilities.

Apart from the abovementioned treatment, APEX‐assisted alleviation of hypoxia can improve radiotherapy. The hollow MnO_2_ loaded with camptothecin (CPT) was coated with a cancer cell membrane to obtain a biomimetic nanozyme/CPT hybrid system.^[^ ^144^ ^]^ After the hybrid system targeting accumulates in the cancer site, not only can MnO_2_ with intrinsic CAT‐mimicking activities decompose H_2_O_2_ into O_2_ to enhance the radiotherapy sensitivity of cancer cells but also CPT can block the cell cycle in the radiosensitive phase. In another case, a CAT@Pt (IV) liposome was designed, which consists of an inner CAT and outer liposome containing cisplatin (IV) prodrug.^[^ ^164^ ^]^ They found that liposomes can maintain enzyme activity, and CAT catalyzes H_2_O_2_ to generate oxygen for enhancing chemotherapy and radiotherapy. Recently, native CAT in *E. coli* has been exploited to develop CAT‐containing *E. coli* membrane vesicles to relieve tumor hypoxia, improve radiotherapy, and induce antitumor immune memory.^[^ ^124^ ^]^ This work illustrated that CAT in bacteria is an effective strategy for cancer treatment.

**Figure 15:**
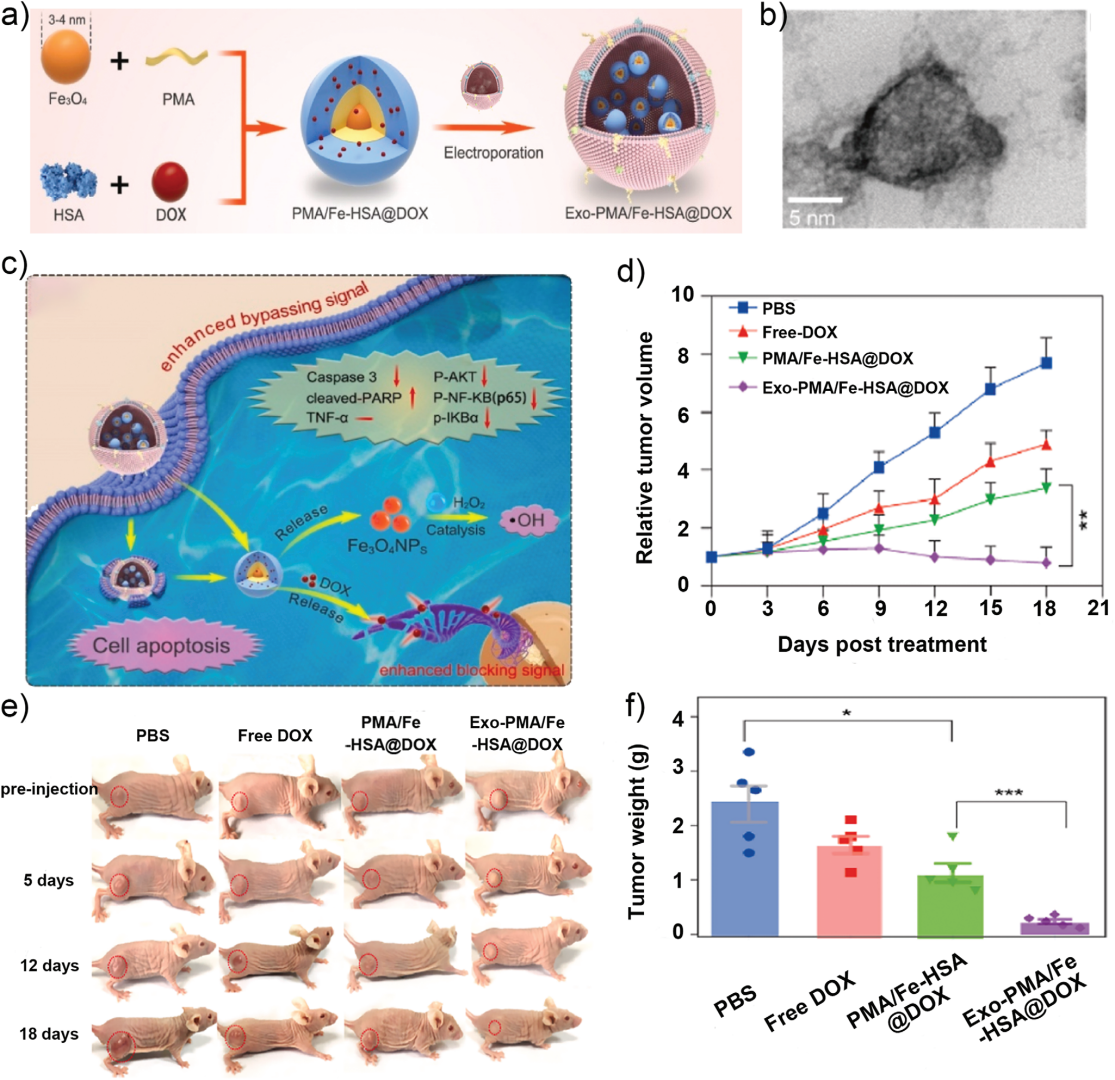
a) Schematic diagram of the formation of Exo‐PMA/Fe‐HSA@DOX NPs. b) TEM image of Exo‐PAM/Fe‐HSA@DOX NPs. c) Schematic diagram of Exo‐PMA/Fe‐HSA@DOX‐targeted prostate cancer therapy. d) Cancer growth curve in different groups. e) Photographs of tumors after the mice received different treatments. f) Tumor volume in the mice in different groups. Reproduced with permission.^[^ ^160^ ^]^ Copyright 2021, Elsevier Ltd.

Additionally, cell membrane‐cloaked catalysts could be used to treat cancer in other ways. For example, a urinary exosome‐based nanovector was fabricated and used for prostate cancer therapy.^[^ ^160^ ^]^ This nanoplatform consists of nano‐sized Fe_3_O_4_ integrated with DOX coated with exosomes from urine (**Figure** **15a**,d). Benefiting from exosome membranes, nanovectors could easily enter homologous cells, followed by Fe_3_O_4_ NPs that exhibit POD‐like activity and catalyze H_2_O_2_ to produce ROS to kill cancer cells directly (Figure 15c), showing prominent synergistic chemodynamic/low‐dose chemotherapy performance in inhibiting cell proliferation (Figure 15d–f).

APEXs have also been used in tumor diagnosis. For the imaging of cancer, Nie and colleagues designed an exosome‐like nanozyme vesicle (FA‐RM: GQDzyme/ABTS) for PAI with the advantage of great spatial resolution and deep tissue penetration.^[^ ^133^ ^]^ Graphene quantum dot nanozyme (GQDzyme) with intrinsic POD‐like activity can convert 2,2′‐azino‐bis (3ethylbenzothiazoline‐6‐sulfonic acid) (ABTS) into oxidized ABTS, which can be used for PAI in the presence of H_2_O_2_. GQDzyme and ABTS were coated by RBC membrane modified with folate acid (FA) that endows vesicles with the ability to target nasopharyngeal carcinoma cells. The nanozyme vesicle efficiently accumulated at cancer sites for cancer diagnosis. The Exo@Au catalysts were prepared with a POD‐like activity using nanozyme‐assisted immunosorbent assay (NAISA).^[^ ^161^ ^]^ Compared with traditional enzyme‐linked immunosorbent assay (ELISA), the original NASIA platform can rapidly analyze different exosome proteins. This work may be a promising method for cancer diagnosis by profiling exosomal proteins from different cells.

### 5.2 Antioxidant and Anti‐Inflammatory Therapies

Elevated intracellular levels of ROS, known as oxidative stress, can cause damage to DNA, proteins, and lipids.^[^ ^172^ ^]^ As a critical signaling molecule, ROS plays an essential role in the progression of inflammation. ROS has been linked to numerous pathologies, including inflammation, tissue injury, and atherosclerosis.^[^ ^173^ ^]^ With the development of nanocatalytic medicine, oxidative stress is a key therapeutic target for many diseases. Some catalysts can be used for antioxidant therapy by removing ROS, but they have not achieved the desired therapeutic response due to insufficiently targeted accumulation. APEXs have been used for antioxidant treatment, which is still in its infancy.

**Figure 16:**
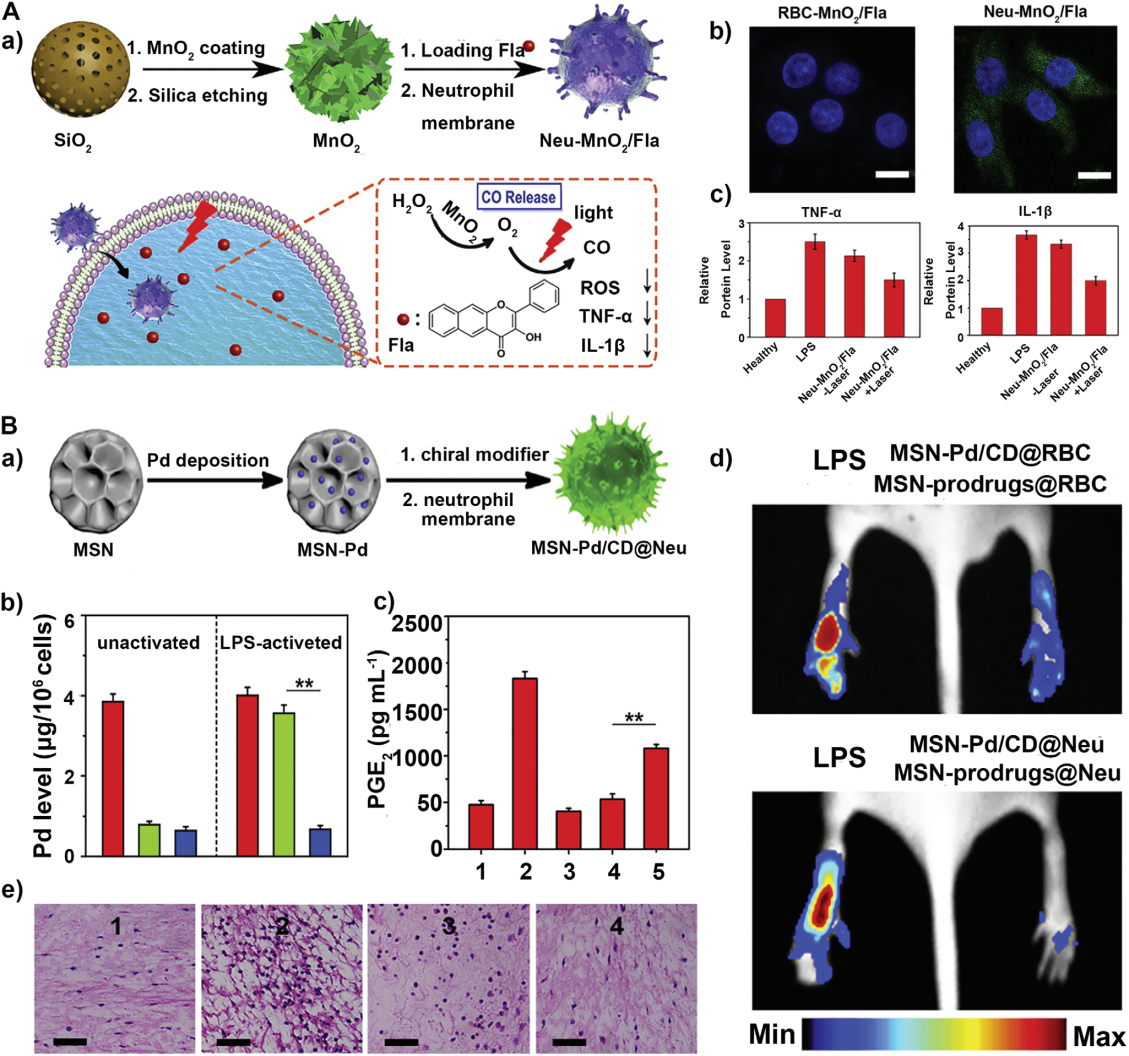
A) a) Schematic diagram of the preparation of Neu‐MnO_2_/Fla and treatment of inflammation in mice. b) Fluorescent images of PC12 cells after incubation with RBC‐MnO_2_/Fla and Neu‐MnO_2_/Fla, respectively. c) Levels of TNF‐*α* and IL‐1*β* of the inflamed paw. Reproduced with permission.^[^ ^156^ ^]^ Copyright 2020, Elsevier Ltd. B) a) Schematic illustration of the preparation of cinchonidine (CD)‐modified catalyst MSN‐Pd/CD@Neu. b) Intracellular Pd levels for MSN‐Pd/CD (red), MSN‐Pd/CD@Neu (green), and MSN‐Pd/CD@RBC (blue) incubated with the unactivated or activated RAW264.7 cells. c) PGE2 level. RAW264.7 cells were treated with (1) blank; (2) LPS; (3) LPS and S‐IBU; (4) LPS, preIBU, HCOONa, and MSN‐Pd/CD@Neu; and (5) LPS, preIBU, HCOONa, and MSN‐Pd/CD@RBC. d) ROS imaging of LPS‐induced inflamed paws. e) H&E staining images of inflamed paws. The rear paws of mice were treated with (1) physiological saline; (2) LPS; (3) LPS, MSN‐Pd/CD@RBC, and MSN‐prodrugs@RBC; and (4) LPS, MSN‐Pd/CD@Neu, and MSN‐prodrugs@Neu. Reproduced with permission.^[^ ^29^ ^]^ Copyright 2020, Elsevier Ltd.

Inflammation is a protective response triggered by harmful stimuli.^[^ ^174^ ^]^ It has been reported that uncontrolled or chronic inflammation causes various diseases, such as cancer, cardiovascular diseases, and diabetes.^[^ ^76^ ^]^ Taking advantage of superfluous H_2_O_2_ in the inflamed tissues, Qu and coworkers prepared a CO‐releasing nanoplatform (called Neu‐MnO_2_/Fla), in which carbon monoxide (CO) shows anti‐inflammatory effects and MnO_2_ can relieve oxidative stress due to the intrinsic CAT activity (Figure 14Aa).^[^ ^156^ ^]^ The Neu‐MnO_2_/Fla was presented by encapsulating flavone (Fla) in MnO_2_ nanoparticles cloaking with a neutrophil cell membrane. On the one hand, compared with the RBC membrane‐wrapped MnO_2_/Fla, Neu‐MnO_2_/Fla has a stronger inflammation targeting ability (**Figure** **16**Ab). On the other hand, Neu‐MnO_2_/Fla reduced the level of pro‐inflammatory cytokines in both in vitro and in vivo models of inflammation (Figure 16Ac). In short, the nanoplatform can realize targeting of the inflammatory site and in situ photoinduced CO release to mitigate tissue inflammation. The same group also reported another neutrophil membrane‐cloaked MSN‐Pd catalyst for treating inflammation.^[^ ^29^ ^]^ The neutrophil membrane was coated on cinchonidine (CD)‐modified Pd, which is fixed on large‐pore MSN to form MSN‐Pd/CD@Neu(Figure 16Ba). With the assistance of a neutrophil membrane, MSN‐Pd/CD@Neu accumulated in inflamed sites to alleviate inflammation in the mouse paw (Figure 16Bb–e). As mentioned above, the encapsulation of the neutrophil membrane gives the nanoparticles the ability to target inflammation. Catalysts coated by cell membranes can be used to treat inflammation.

### 5.3 Neuron Protection

**Figure 17:**
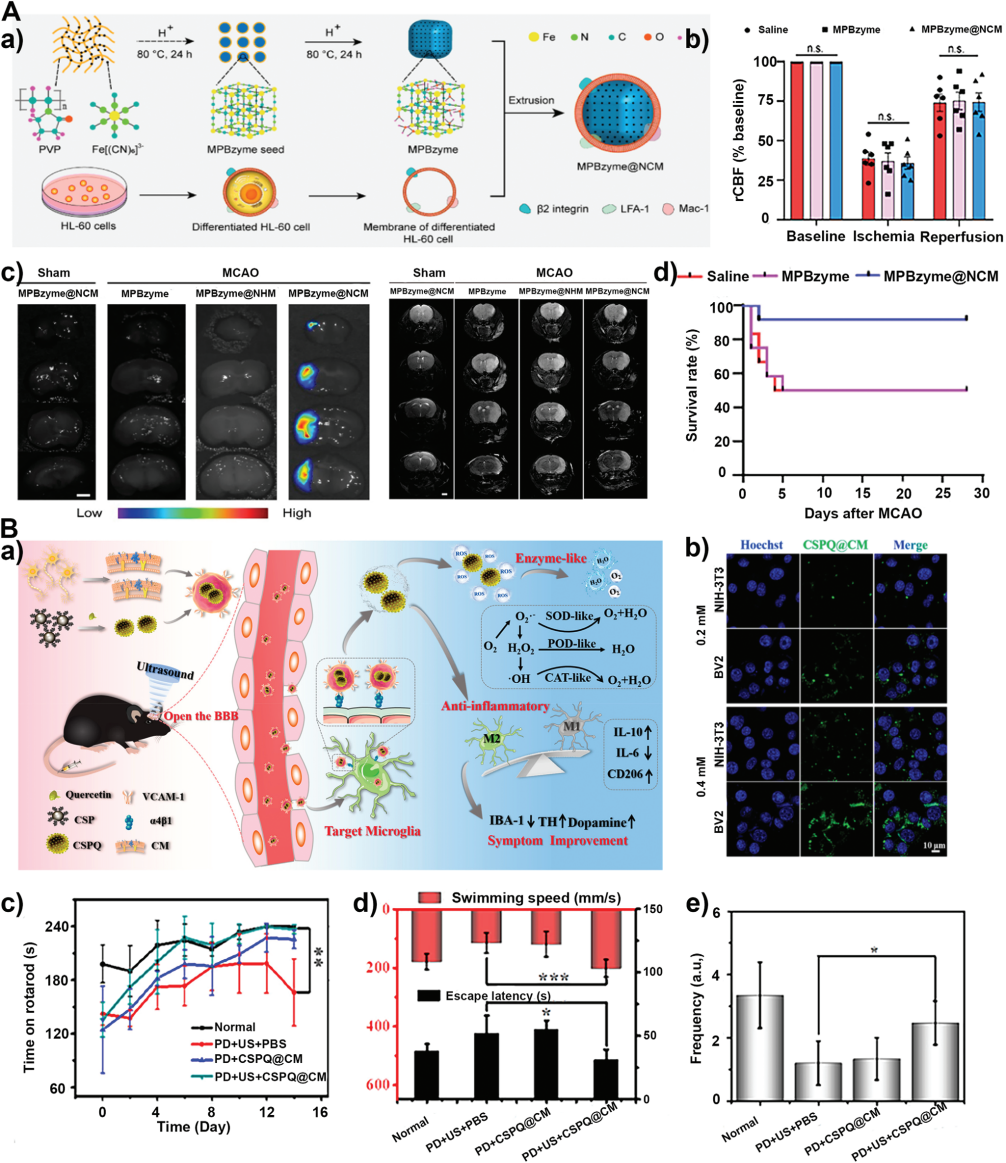
A) a) Scheme of the MPBzyme@NCM synthesis process. b) rCBF in each group. c) Images of brain coronal sections and axial view of T2‐weighted magnetic resonance images 24 h after intravenous administration of FITC‐labeled MPBzyme@NCM, MPBzyme, or MPBzyme@NHM. d) Survival rate 28 days after transient middle cerebral artery occlusion (tMCAO)/stroke from differently treated mice. Reproduced with permission.^[^ ^152^ ^]^ Copyright 2019, Wiley‐VCH. B) a) Schematic illustration of biomimetic CSPQ@CM nanoparticles for treatment of Parkinson's disease. b) CLSM images and in NIH‐3T3 and BV2 cells after incubation with different concentrations of CSPQ@CM nanoparticles. c) Rotatory‐rod test, d) swimming speed and escape latency, e) frequency of occurrence in the target quadrant of normal mice, Parkinson's disease (PD) mice treated with PBS, and PD mice treated with/without focused ultrasound before intravenous injection of CSPQ@CM nanoparticles. Reproduced with permission.^[^ ^176^ ^]^ Copyright 2020, American Chemical Society.

Ischemic stroke, an acute and severe disease, can lead to disability and death. Blood vessel occlusion results in a sudden cut‐off of circulation in a part of the brain. Timely thrombolysis is the main treatment for ischemic stroke.^[^ ^175^ ^]^ After thrombolysis, oxidative stress during ischemia and reperfusion leads to damage to brain tissue. It has been reported that free radical scavenging is a promising strategy for treating ischemic stroke.^[^ ^132^ ^]^ Various catalysts with antioxidase activity have been used for free radical scavenging. As discussed in Section 3.3, the major impediment to the treatment of brain diseases is the existence of BBB. A novel drug delivery strategy was developed, cellular backpacks, for neurodegenerative disorders.^[^ ^157^ ^]^ The CAT backpack is loaded with macrophages, which cross the BBB via natural recruitment during inflammation. CAT, one of the most potent antioxidants, can attenuate free radical production for treating neurodegenerative diseases. Similarly, the work by Liu et al. is worth noting as they proposed an engineered nanoerythrocyte (MNET) that can cross BBB due to the native stealth property and assisting of erythrocyte membrane modified by T7 peptide.^[^ ^132^ ^]^ Because of the Mn_3_O_4_ core, MNET exhibited free radical scavenging ability. Interestingly, MNET with natural oxygen sponge effect not only scavenges free radicals but also possesses the ability to supply oxygen timely before thrombolytic therapy to save nerve cells and store oxygen after thrombolytic therapy. With these abilities, MNET significantly attenuated neurological damage. Such MNET is vital for reducing free radicals and displays a clinical application for an ischemic stroke. Additionally, after ischemic stroke, neurological functional recovery is hindered by oxidative stress and excessive inflammatory responses. To make catalysts accumulate in the ischemic brain, a targeted nanozymes delivery platform was reported (**Figure** **17**Aa).^[^ ^152^ ^]^ Because of the expression of Mac‐1, expression of integrin *β*2, and lymphocyte function‐associated antigen 1 on the neutrophil membrane, neutrophils can interact with inflamed brain microvascular endothelial cells. And human promyelocytic leukemia cell membranes with neutrophil membrane properties are used to encapsulate mesoporous Prussian blue nanozyme to realize the targeting delivery of MPBzyme@NCM to the damaged brain (Figure 17Ab). In this nanoplatform, MPBzyme is a ROS scavenger to treat ischemic brain injury and restore neurological function (Figure 17Ac,d). This work provides a novel strategy for catalysts to enter the brain parenchyma.

Neurodegenerative diseases are one of the most common diseases, which are caused by the degeneration or death of the structure or function of neurons.^[^ ^177^, ^178^ ^]^ Alzheimer's disease, which is defined by amyloid‐*β* (A*β*) plaques and neurofibrillary tangles, is the most common form of dementia in the elderly.^[^ ^179^, ^180^ ^]^ A bioinspired antioxidant nanozyme coated with A*β*‐targeting peptide‐modified erythrocyte membrane (Cu*_x_* O@EM‐K) was designed.^[^ ^137^ ^]^ The Cu*_x_* O core exhibits excellent multiple antioxidant enzyme‐like activities. A*β*‐targeting pentapeptide KLVFF modified erythrocyte membranes with both specific targeting ability and low immunogenicity. Cu*_x_* O@EM‐K can be the selective and effective clearance of peripheral A*β* and reverse learning and memory impairments in 3xTg‐AD mice. Microglia, as an important target for the treatment of various neurodegenerative diseases, has aroused wide attention. For example, researchers proposed neuronal cell membrane‐coated biomimetic nanoparticles (called CSPQ@CM) that can target microglia to treat PD.^[^ ^176^ ^]^ CSPQ has multiple enzyme activities toward scavenging ROS. Importantly, CSPQ@CM promotes microglia to enter the M2‐like phenotype with anti‐inflammatory properties (Figure 17Ba). As indicated by in vitro and in vivo experiments, due to the encapsulation of neuron cell membranes, CSPQ nanoparticles were more likely to be phagocytosed by BV2 cells (Figure 17Bb). This work demonstrated that CSPQ@CM could relieve the symptoms of PD and improve cognition impairment and dyskinesia in PD mice (Figure 17Bc–e).

## 6 Conclusion and Perspective

APEXs have outstanding biocompatibility, immune escape ability, and unique catalytic characteristics due to the combination of membrane and catalysts. Different from bare catalysts, APEXs achieve a fresh design based on catalysts inspired by biology. On the one hand, catalysts are more stable than natural enzymes, and their activity can be regulated. On the other hand, membrane and membrane proteins further give some unique biological properties to catalysts. In this review, we reviewed the strategies for designing APEXs in recent years and their development in biomedical. We also outlined the extraction of membranes and the characteristics of various membranes, which can help to understand the design ideas of membrane‐cloaked and its advantages in diverse application fields.

**Figure 18:**
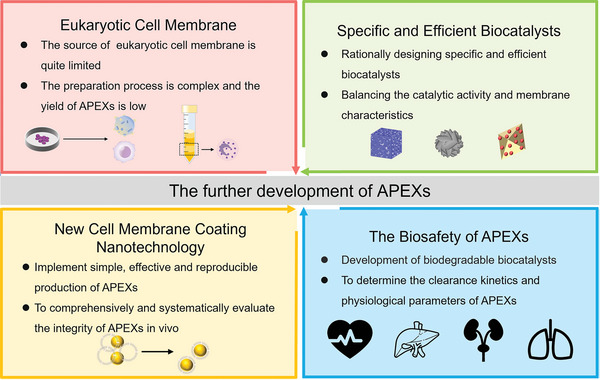
Illustration of the further development of APEXs.

Although a variety of membrane‐cloaked catalysts has been developed and used for biomedical applications, substantial potential challenges, the source of the membrane, and techniques of membrane extraction and coating have prevented clinical applications for a short period. We will discuss the further development of APEXs from the perspective of fundamental research (**Figure** **18**).

1) Searching for a more stable source of the eukaryotic cell membrane and exploring a simpler method to extract the eukaryotic cell membrane. It is well known that the source of eukaryotic cells is limited, and the procedure of membrane extraction is more complicated, which will limit the yield of APEXs. Although artificial membranes such as liposomes can be synthesized, proteins on natural membranes can give APEXs more properties, such as immune escape and homologous targeting. Therefore, exploring simpler methods to extract eukaryotic cell membranes is necessary.
2) Rationally designing specific and efficient catalysts for boosting the biomedical applications of APEXs. Many types of research have shown that the structure of catalysts, such as size, morphology, composition, and surface charge, is the key factor affecting the catalytic activities. However, considering the coating of the membrane on the surface catalysts, it may be necessary to discard some physicochemical properties of catalysts or combine natural enzymes to better balance the catalytic activity and membrane characteristics to achieve specific and efficient biological applications.
3) Exploring new cell membrane coating nanotechnology for simple, effective, and reproducible production of APEXs. The typical membrane coating technique is extrusion, which results in the loss of the shell and core of APEXs. In addition, the integrity of the cell membrane and the core of the catalyst should be maximally retained to keep the functionality during blood circulation. To address this issue, a comprehensive and systematic approach to in vivo integrity assessment of APEXs would be attractive.
4) Systematically and comprehensively evaluating the biosafety of APEXs. After entering the body, APEXs will accumulate in healthy tissues and organs inside the body. Although APEXs have higher biocompatibility than bare catalysts, biosafety is still a key issue that needs to be carefully considered at the design and application stage of APEXs, such as the development of biodegradable catalysts to reduce the long‐term accumulation of APEXs in the body. However, the determined clearance kinetics and physiological indexes also need a comprehensive analysis, where the effect of both catalysts and membranes needs to be addressed.

In summary, APEXs are a promising biomedical applications strategy with rapid development. What we describe is just their latest applications in biomedical areas. We also believe that with the joint efforts of researchers from multiple disciplines, the above technical and biosafety issues will be gradually solved, promoting the safe and efficient application of APEXs in the biomedical field. Moreover, we expect this progress review provides promising perspectives and broadens the routes to designing bioinspired APEXs, which may propel membrane‐cloaked catalytic applications.

## Conflict of Interest

The authors declare no conflict of interest.
